# Current phenotypic and genetic spectrum of syndromic deafness in Tunisia: paving the way for precision auditory health

**DOI:** 10.3389/fgene.2024.1384094

**Published:** 2024-04-22

**Authors:** Rahma Mkaouar, Zied Riahi, Jihene Marrakchi, Nessrine Mezzi, Lilia Romdhane, Maroua Boujemaa, Hamza Dallali, Marwa Sayeb, Saida Lahbib, Hager Jaouadi, Hela Boudabbous, Lotfi Zekri, Mariem Chargui, Olfa Messaoud, Meriem Elyounsi, Ichraf Kraoua, Anissa Zaouak, Ilhem Turki, Mourad Mokni, Sophie Boucher, Christine Petit, Fabrice Giraudet, Chiraz Mbarek, Ghazi Besbes, Soumeyya Halayem, Rim Zainine, Hamida Turki, Amel Tounsi, Crystel Bonnet, Ridha Mrad, Sonia Abdelhak, Mediha Trabelsi, Cherine Charfeddine

**Affiliations:** ^1^ Laboratory of Biomedical Genomics and Oncogenetics LR16IPT05, Pasteur Institute in Tunis, University of Tunis El Manar, Tunis, Tunisia; ^2^ Department of Otorhinolaryngology, District Hospital of Menzel Bourguiba, Bizerte, Tunisia; ^3^ Department of Biology, Faculty of Sciences of Bizerte, Université Tunis Carthage, Tunis, Tunisia; ^4^ Genetic Typing Service, Institut Pasteur of Tunis, Tunis, Tunisia; ^5^ Marseille Medical Genetics (MMG) U1251, Aix Marseille Université, INSERM, Marseille, France; ^6^ Department of Pediatrics, La Rabta Hospital, Tunis, Tunisia; ^7^ Laboratory of Hereditary Diseases of the Metabolism Investigation and Patients Management, Faculty of Medicine in Tunis, University of Tunis El Manar, Tunis, Tunisia; ^8^ Department of Epidemiology and Public Health, Directorate General of Military Health, Faculty of Medicine in Tunis, University of Tunis El Manar, Tunis, Tunisia; ^9^ ICHARA Association (International Research Institute on Sign Language), Tunis, Tunisia; ^10^ Department of Congenital and Hereditary Diseases, Charles Nicolle Hospital in Tunis, Tunis, Tunisia; ^11^ LR99ES10 Laboratory of Human Genetics, Faculty of Medicine in Tunis, University of Tunis El Manar, Tunis, Tunisia; ^12^ Child and Adolescent Neurology Department of Neurology, National Institute of Neurology, Tunis, Tunisia; ^13^ LR18SP04 Department of Child Neurology, National Institute Mongi Ben Hmida of Neurology in Tunis. University of Tunis El Manar, Tunis, Tunisia; ^14^ Department of Dermatology, Habib Thameur Hospital, Research Unit Genodermatoses and Cancers LR12SP03, Tunis, Tunisia; ^15^ Service de dermatologie, Hôpital La Rabta, Unité de recherche UR 12SP07, Hôpital La Rabta, Tunis, Tunisia; ^16^ Service d’ORL et chirurgie cervico-faciale, CHU d’Angers, Angers, France; ^17^ Equipe Mitolab, Institut Mitovasc, CNRS UMR6015, UMR Inserm 1083, Université d’Angers, Angers, France; ^18^ Institut Pasteur, Université Paris Cité, Inserm UA06, Institut de l’Audition, Paris, France; ^19^ Collège de France, Paris, France; ^20^ Unité Mixte de Recherche (UMR) 1107, INSERM, Clermont-Ferrand, France; ^21^ Centre Auditif SoluSons, Clermont-Ferrand, France; ^22^ ENT Department, Habib Thameur Hospital, Tunis, Tunisia; ^23^ Department of Otorhinolaryngology and Maxillofacial Surgery - La Rabta Hospital in Tunis, Tunis, Tunisia; ^24^ Service de pédopsychiatrie, Hôpital Razi, Faculté de Médecine de Tunis, Université Tunis el Manar, Tunis, Tunisia; ^25^ Dermatology Department Hedi Chaker University Hospital, Sfax University Sfax Tunisia, Tunis, Tunisia; ^26^ CNSS Polyclinic, Bizerte, Tunisia

**Keywords:** syndromic deafness (SD), spectrum of SDs, next-generation sequencing (NGS), early detection, under-diagnosis, Tunisia

## Abstract

Hearing impairment (HI) is a prevalent neurosensory condition globally, impacting 5% of the population, with over 50% of congenital cases attributed to genetic etiologies. In Tunisia, HI underdiagnosis prevails, primarily due to limited access to comprehensive clinical tools, particularly for syndromic deafness (SD), characterized by clinical and genetic heterogeneity. This study aimed to uncover the SD spectrum through a 14-year investigation of a Tunisian cohort encompassing over 700 patients from four referral centers (2007–2021). Employing Sanger sequencing, Targeted Panel Gene Sequencing, and Whole Exome Sequencing, genetic analysis in 30 SD patients identified diagnoses such as Usher syndrome, Waardenburg syndrome, cranio-facial-hand-deafness syndrome, and H syndrome. This latter is a rare genodermatosis characterized by HI, hyperpigmentation, hypertrichosis, and systemic manifestations. A meta-analysis integrating our findings with existing data revealed that nearly 50% of Tunisian SD cases corresponded to rare inherited metabolic disorders. Distinguishing between non-syndromic and syndromic HI poses a challenge, where the age of onset and progression of features significantly impact accurate diagnoses. Despite advancements in local genetic characterization capabilities, certain ultra-rare forms of SD remain underdiagnosed. This research contributes critical insights to inform molecular diagnosis approaches for SD in Tunisia and the broader North-African region, thereby facilitating informed decision-making in clinical practice.

## 1 Introduction

Hearing Impairment (HI) is the most frequent sensorineural disorder worldwide. HI is characterized by a broad spectrum of clinical manifestations which allows its classification, depending on the age of disease onset, the location (conductive, sensorineural, mixed), and the severity (mild, moderate, severe, profound) of the HI. The prevalence of bilateral sensorineural HI has been estimated to be 1 per 500–1,000 newborns (Ideura et al., 2019; Bussé et al., 2020). The incidence of mild HI has been estimated to be 1 per 750 infants in developed countries (Zazo Seco et al., 2017). Severe to profound HI represents 35% of affected cases (Boudewyns et al., 2018). Genetic etiologies account for over half of all congenital HI cases (Calvet et al., 2018; Ideura et al., 2019). The remainder are associated with non-genetic factors such as ototoxic drugs and, materno-fetal infection including cytomegalovirus (CMV) infection.

It has been shown that more than 250 genes, accounting for 1% of the human coding genome, are involved in the development and functioning of the auditory system (Sheppard et al., 2018). Around 6% of rare diseases affect hearing. Genetic HI has been studied in the Tunisian population for several years. The two first Autosomal Recessive Non-Syndromic Hearing Loss (ARNSHL) loci (DFNB1 and DFNB2) were discovered for the first time by Guilford et al. in 3large consanguineous families originating from Northern andSouthern-Tunisia (Guilford et al., 1994). However, there is a considerable lack of epidemiological data and a complete absence of specific registries. Upon the last available epidemiological data in a survey conducted by Ben Arab et al., the prevalence of congenital HI has been estimated to be 0.32% (Ben Arab et al., 2004).

HI can also be classified according to the absence or presence of additional clinical features into Non-Syndromic Hereditary Hearing loss (NSHHL) and Syndromic Deafness (SD), respectively. Diseases associated with HI in SD can affect one or more organ systems, namely, the eye, skin, thyroid, kidney, heart, and nervous system. SD accounts for approximately 30% of congenital HI cases. The prevalence of SDs has been estimated to be 3/10,000 live births (Gettelfinger and Dahl, 2018). The London Dysmorphology database reports roughly 400 SDs identified worldwide (Castiglione et al., 2013).

Among the most frequent SDs worldwide, we can mention Pendred syndrome (PDS), Usher syndrome (USH), and Waardenburg syndrome (WS). PDS (# 274600) is globally known as the first and most frequent deafness syndrome accounting for up to 10% of all the genetic forms of HI (Diramerian and Ejaz, 2023). It is an autosomal recessive disorder described for the first time by Vaughan Pendred in 1896 (Gettelfinger and Dahl, 2018). In the general population, PDS is a rare condition with a prevalence of 7.5-10/100,000 cases. This disorder is characterized by sensorineural HI associated with inner ear malformations and a thyroid dysfunction manifested by goiter.

The most common related gene is *SLC26A4* (OMIM 605646) which is also the second most common cause of ARNSHL (DFNB4). This gene encodes for pendrin which is a transmembrane anion transporter highly expressed in the inner ear, thyroid, and kidney (Gettelfinger and Dahl, 2018; Tesolin et al., 2021).

USH is the second most frequent SD with a prevalence estimated between 3.2 and 6.2/100,000 cases depending on the population (Fuster-García et al., 2021). The syndrome is defined as the association of HI, vestibular dysfunction, and progressive loss of vision leading to blindness, termed ‘ Retinitis Pigmentosa’ (RP). Loss of night vision and narrowing of the visual field are usually considered as the primary signs consistent with RP (Ivanova et al., 2018). Vision impairment worsens the gait disturbances resulting from the vestibular dysfunction (El-Amraoui and Petit, 2014; Gettelfinger and Dahl, 2018). The USH phenotype is conventionally subdivided into three clinical subtypes. USH1, which is the most frequent clinical subtype, is characterized by HI and RP developed during infancy or adolescence and marked by its association with vestibular dysfunction. This latter often appears as an inability to walk and sit before the age of 18 months. The onset of USH syndrome in clinical subtype II (USH2) occurs in adolescence or adulthood without any signs of vestibular disorder. USH3 is marked by progressive HI that appears in late childhood or around puberty stage with or without a vestibular disequilibrium (Fuster-García et al., 2021). In all three clinical subtypes, RP could be accompanied by cataracts (Gettelfinger and Dahl, 2018). The clinical subtype IV refers to atypical USH phenotypes that do not fit any of the three conventional clinical categories (Fuster-García et al., 2021). To date, 10 major genes have been associated with the three typical clinical forms of USH, thereby revealing the genetic and mutational heterogeneity of this syndrome. *MYO7A, USH1C, CDH23, PCDH15, and SANS* genes are involved in USH1. Three genes have been associated with USH2, namely, *USH2A, ADGVR1, and WHRN*. USH3 is induced by mutations in the *CLRN1* gene. The aforementioned genes encode proteins expressed in the hair cells of the inner ear as well as the photoreceptors of the retina (Jouret et al., 2019; Géléoc and El-Amraoui, 2020; Delmaghani and El-Amraoui, 2022). USH1-associated proteins, expressed at the tips of the stereocilia, are all involved in the development of the hair bundle (Géléoc and El-Amraoui, 2020). USH2 proteins are expressed transiently during embryonic development, 1–2 days after USH1 proteins. Their role is to refine the V shape of the hair bundle (Delmaghani and El-Amraoui, 2022).

Despite its rarity, Waardenburg syndrome (WS) is the third most common form of SD worldwide and represents 1%–3% of all SD forms identified to date (Ahmed Jan et al., 2021). The prevalence and incidence of WS have been estimated to be 1 per 20,000–40,000 and 1 to 2 per 8,400, respectively (Ideura et al., 2019). The syndrome was described for the first time in 1951 by the Dutch ophthalmologist and geneticist Petrus Johannes Waardenburg. It is characterized by the association of HI with pigmentation abnormalities of the eye, hair, and skin (Koffler et al., 2015). Such a condition affects melanocytes in different body tissues, namely, the stria vascularis of the cochlea. WS is classified into four clinical subtypes depending on the presence or absence of additional symptoms such as dystopia canthorum that characterizes WS types I and III, musculoskeletal malformations of the upper limbs in type III and Hirschprung disease in type IV. Six genes are known to be implicated in WS, including *PAX3, MITF, SOX10, SNAI2, EDN3*, and *EDNRB* with 284 mutations described to date. A dominant mode of inheritance is often considered as the major criterion of WS (Huang et al., 2021).

To the best of our knowledge, we report here, for the first time in a MENA (Middle East and North-Africa) country, the spectrum of SD in Tunisia following the clinical, genetic, and mutational investigations performed in a large cohort as well as a literature review of the reported SD forms in the Tunisian population.

## 2 Materials and methods

### 2.1 Patients recruitment

A total of 700 patients were recruited at 4 major referral centers for HI in Tunisia, namely, the Ear-Nose-Throat Department at La Rabta Hospital in Tunis, the Department of Congenital and Hereditary Diseases at Charles Nicolle Hospital in Tunis, the Department of Pediatrics at La Rabta Hospital in Tunis, and the Department of Neuropediatrics at the Mongi BEN HMIDA Institute of Neurology in Tunis. Patients were enrolled in our study if they met at least one of the following criteria: Family history of HI, parental consanguinity, presence of HI associated with other anomalies, and exclusion of environmental HI etiologies. Patients presenting with syndromic deafness attributed to chromosomal rearrangements, including conditions such as Down syndrome or Klinefelter syndrome, were excluded from the study cohort based on thorough review of their clinical records at the designated referral centers as well as clinical examination.

We decided to include in the study cohort both SD and NSHHL cases considering that previous studies in the world (Xiang et al., 2022) and in Tunisia (Riahi et al., 2015) have shown that apparently NSHHL patients harbored variants in genes associated with SD. Such findings have led to reexamining the patients and discovering the overlooked anomalies or additional clinical signs not initially recorded, thus confirming a syndromic condition.

### 2.2 Methods

#### 2.2.1 Clinical evaluation

Audiological assessments, conducted at the ENT department at La Rabta hospital in Tunis, included both subjective (visual reinforcement audiometry (for children), pure-tone audiogram, speech audiometry) and objective tests (tympanometry, middle-ear muscle reflex, otoacoustic emissions, auditory brainstem responses). Detection of inner ear malformations was carried out by temporal bones tomography and Magnetic Resonance Imaging (MRI) of the inner ear. Complementary clinical explorations including ocular fundus examination, cardiac and renal echography were systematically performed.

#### 2.2.2 Genetic screening

##### 2.2.2.1 Genetic screening strategy

For individuals initially diagnosed with apparently non-syndromic HI, our study employed Sanger sequencing as the primary method targeting the *GJB2* gene, since it is the most frequently associated with genetic HI. Furthermore, we extended the sequencing to encompass other genes in families where specific causal variants were identified following a cascade screening approach. In cases where Sanger sequencing did not yield conclusive results or for patients clinically confirmed with syndromic deafness (SD), Targeted Gene Sequencing (TGS) was used for a comprehensive analysis of genes associated with both non-syndromic and specific syndromic forms of SD. In instances where TGS did not provide definitive outcomes or for clinically undiagnosed cases of SD, Whole Exome Sequencing (WES) was performed for a comprehensive screening of genes linked to both non-syndromic and non-specific forms of SD, particularly atypical and ultra-rare conditions.

Among the 700 patients enrolled in this study, molecular analyses were conducted on 554 cases (78.71%) diagnosed with either non-syndromic HI or SD. Sanger sequencing was used for genetic testing in 424 patients. Additionally, 118 patients were subjected to TGS, while twelve patients underwent WES.

##### 2.2.2.2 Targeted gene sequencing (TGS)

Genomic DNA was extracted from peripheral blood leukocytes using standard protocols. A targeted gene sequencing of 113 HI-related loci was carried out in each patient. Genomic DNA capture, sequencing and variant calling were conducted by IntegraGen S.A (Evry, France). Library preparation was performed by NEBNext Ultrakit (New England Biolabs). For each sample, genomic DNA was fragmented by sonication to produce 150–200 db fragments. Purified DNA fragments libraries were hybridized to the SureSelect oligo probe capture library for 72 h followed by washing and elution. The eluted fraction was qPCR -amplified and quantified by qPCR. Quantification enabled the creation of equimolar pools that were again quantified by qPCR. Sequencing of 150 pb paired-end reads was performed on an Illumina HiSeq 2,500.

Bioinformatic analysis of sequencing raw data was conducted by an in-house pipeline. Alignment of sequencing reads to the reference genome (hg19) was carried out using the Burrows-Wheeler Aligner-MEM software (BWA-MEM version 1.1.1; https://bio-bwa.sourceforge.net). Variant calling was performed using the Genome Analysis Tool Kit (GATK) algorithm HaplotypeCaller.

##### 2.2.2.3 Whole exome sequencing (WES)

Exon capture was performed using the Agilent SureSelect XT Human All Exon v6 (60 Mb) Version C2 (December 2018). Following library preparation, samples were sequenced on a NovaSeq6000 platform (Illumina, San Diego, California) with a 150 bp paired-end reads configuration. Raw sequence files were aligned to the human genome reference sequence (version hg19) using the BWA-MEM software (BWA-MEM version 1.1.1; https://bio-bwa.sourceforge.net). Duplicates removal from BAM files was performed using the PICARD tool (www.picard.sorceforge.net). The GATK tool (GATK, www.broadinstitute.org/gatk/) was used for Indels realignment by the Realigner Target Creator algorithm. GATK was also used for base quality score recalibration. Variant Calling was performed by the GATK package Haplotype Caller.

##### 2.2.2.4 Variant annotation and prioritization

For both TGS and WES data, variant annotation and prioritization were conducted using the VarAFT software version 2.16 (http://varaft.eu/) (Desvignes et al., 2018). SNPs and INDELs located in functionally relevant genomic regions, .i.e., exons and splice sites, were selected. Variants identified as synonymous or non-coding were excluded. Sequencing coverage statistics were calculated using the VarAFT tool. Genetic variants, with a read depth<20X and a mapping quality score<30, were discarded. A frequency filter was then applied to only include variants with a Minor Allele Frequency (MAF) threshold of 0.1 or less according to 1000Genomes (https://www.internationalgenome.org/), gnomeAD (https://gnomad.broadinstitute.org/), and GME (Greater Middle East) Variome (http://igm.ucsd.edu/gme/) databases.

The functional effects of genetic sequence variants were assessed by *in silico* prediction tools including SIFT (sift.bii.a-star.edu.sg/), MutationTaster (mutationtaster.org/); PolyPhen (genetics.bwh.harvard), FATHMM (http://fathmm.biocompute.org.uk), PROVEAN v1.1 (provean.jcvi.org/), MutationAssessor 1.0 (mutationassessor.org/r3/), and VarSome (https://varsome.com). Genetic variants, for which the CADD-Phred score is less than 15, were filtered out.

##### 2.2.2.5 Copy number variation (CNV) detection and analysis

CNVs were called from TGS data using the ExomeDepth R package that detects CNVs from targeted sequencing data based on a read depth approach. The alignment data (BAM file) of the tested patient were compared to a matched aggregate reference set that combines five control BAM files generated by identical NGS sequencing and bioinformatic analysis procedures.

##### 2.2.2.6 Sanger sequencing

Polymerase Chain Reaction (PCR) was performed on genomic DNA samples using primers designed by the primer3 software. PCR products were sequenced using the BigDye terminator v3.1 cycle sequencing reaction kit on an ABI prism 3130 DNA Genetic Analyzer (Applied Biosystems, Foster City, CA, United States) in accordance with the manufacturer’s recommendations. The ABI files were analysed using the BioEdit sequence alignment editor (version 7.2).

#### 2.2.3 Literature data extraction

We performed a systematic and comprehensive search of the published literature until February 2022. The bibliographic data sources included scientific publications available in Pubmed (https://pubmed.ncbi.nlm.nih.gov/), Scopus (https://www.scopus.com/), Web of Science (https://www.webofscience.com), Science direct (https://www.sciencedirect.com), and OMIM (https://www.omim.org/) databases. In order to gather all available studies on SD, broad search terms such as ‘Syndromic hearing impairment’ AND ‘Tunisia’ were used. The names of frequent deafness syndromes were also used as keywords. Articles reporting either clinical descriptions, genetic studies or both were captured. We also referred to the local genetic diseases database, developed at the LGBMO and gathering 589 entities (Mezzi et al., 2021), in order to retrieve genetic conditions associated with HI. Grey literature sources included medical and scientific dissertations or theses, abstracts and posters presented in national and international conferences as well as those found in websites and specialized databases.

## 3 Results

### 3.1 Preliminary clinical classification of the studied cohort

A total of 700 patients belonging to 620 families have been enrolled in the current study. Among these families, 221 (35.64%) were multiplex, i.e., more than one affected case in the nuclear and/or the extended family.

SDs identified in the studied cohort have been classified into two major clinical categories based on the patient phenotype. The first one corresponds to specific syndromes that include well known clinical entities that are defined as SDs in the literature and reported in HI specific databases. The second category includes non-specific syndromic forms, multisystemic diseases, and atypical phenotypes in which HI is a secondary or an uncommon clinical manifestation. Comorbidities are also included in this disease group.

The studied cohort has been categorized into 4 clinical subsets; i) Patients affected with apparently non-syndromic deafness; ii) Patients presenting with specific SDs; iii) Patients presenting with non-specific SDs; iv) Patients with suspected comorbidities.

Clinical data collection has allowed us to classify 78 patients, representing 11% of the total cohort, as having a SD. These patients belonged to 70 families, among whom 30 were consanguineous (38.46%) and 20 were multiplex. The age range varied between 4 and 75 years. Sixteen SDs were identified; five of which corresponded to specific clinical forms, including Waardenburg syndrome (WS), Alport syndrome, Treacher-Collins syndrome, Usher syndrome (USH), and CHARGE syndrome (Coloboma, Heart Anomaly, Choanal Atresia, Retardation, Genital, and Ear Anomalies). Twelve non-specific syndromic forms were identified following our census.

Two comorbidities were suspected in two patients; the first one associates unilateral Hearing Loss (HL) with triple A syndrome (or Allgrove syndrome) and the second one combines deafness with Moyamoya disease. The two patients with these comorbidities are from first degree consanguineous marriages, a factor that favors the co-occurrence of two or more disorders.

### 3.2 Molecular investigation of SDs in the studied cohort

**FIGURE 1:**
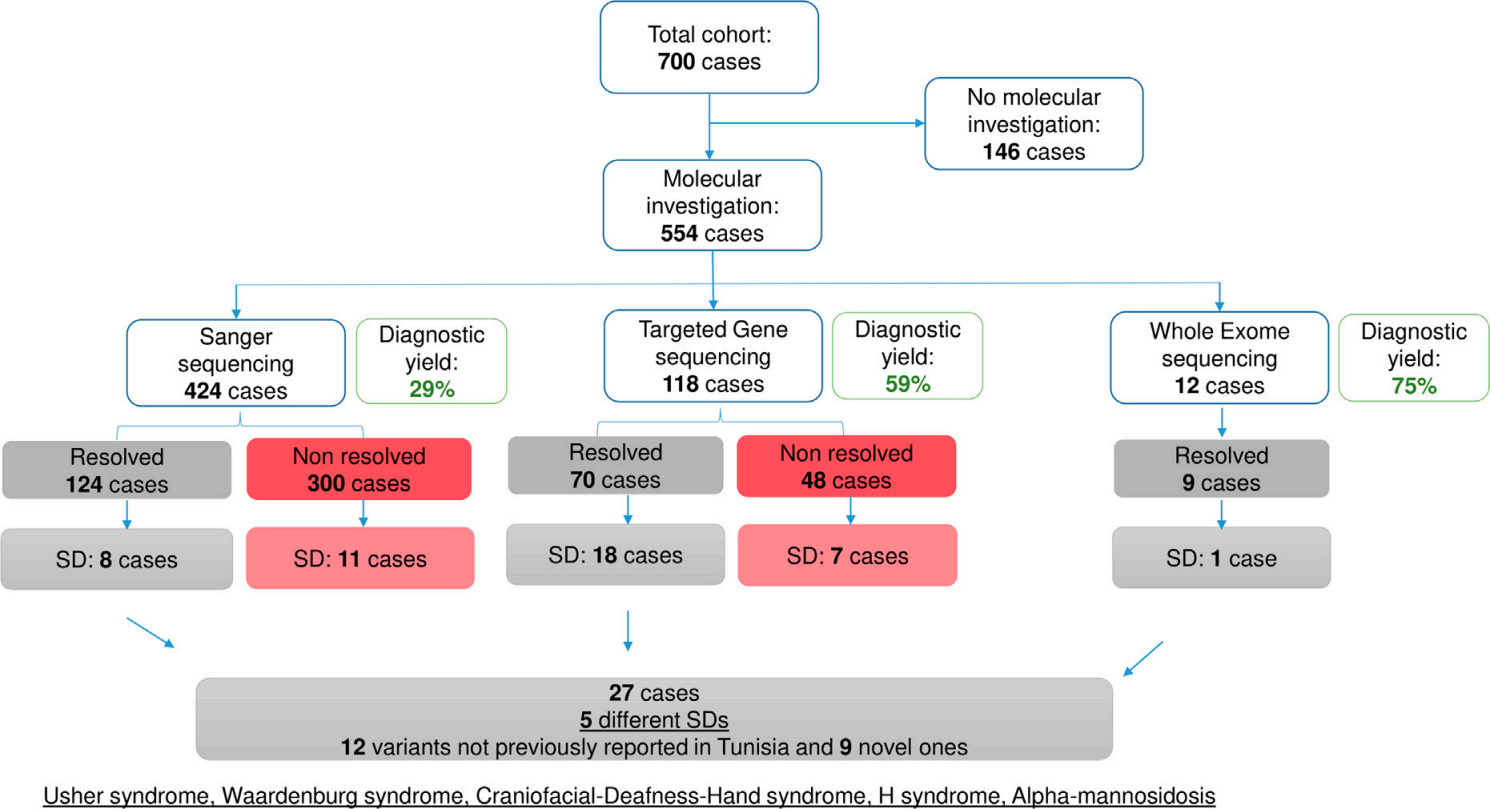
Overview of the molecular investigation in the studied cohort. Diagram summarizing the molecular investigation carried out in the studied cohort using 3 approaches: Sanger sequencing, Targeted Gene Sequencing and Whole Exome Sequencing. Diagnostic yields are indicated.

Sanger sequencing confirmed non-syndromic HI, whether associated with the *GJB2* gene or not, in 116 patients (21% of the investigated cases (216/424); 57% of the resolved cases), and SD in eight patients. TGS identified the molecular etiology in 70 cases out of the 118 patients tested, with 18 presenting with SD. Exome analysis conducted in twelve patients revealed SD in only one case, corresponding to alpha-mannosidosis (AM), an ultra-rare SD reported for the first time in the Tunisian population Figure 1.

Following the molecular investigation, five syndromic clinical entities, including USH, WS, craniofacial-deafness-hand syndrome (CFDH), H syndrome (HS), and AM were identified in 27 patients. The AM cases have already been published elsewhere (Mkaouar et al., 2021) Figure 1.

#### 3.2.1 Genetic investigation of usher syndrome (USH)

The diagnosis of USH was strongly suggested in five patients (DF202, DF252, DF522, DF618, DF619), in the presence of a HI associated with retinitis pigmentosa (RP) that was revealed by electroretinography (ERG) and Optical Coherence Tomography (OCT) (Table 1). USH was also suspected in the patient DF211 given that he had a history of walking delay, which suggested a vestibular dysfunction. The remaining four cases were initially diagnosed as non-syndromic HI.

**TABLE 1:** Clinical characteristics of the patients presenting with Usher syndrome.

Patient code	Age (years)	Hearing impairment degree	Associated clinical features
DF252	8	Bilateral profound congenital (110 dB)	RP, psychomotor delay, brain MRI: subcortical atrophy with diffuse and homogeneous moderate hypoplasia of the corpus callosum
DF202	9	Bilateral profound congenital	RP, psychomotor retardation, cerebral palsy, axial hypotonia, quadri-pyramidal syndrome, strabismus, nystagmus
DF211	9	Bilateral profound	RP, walking delay
DF159	11	Bilateral profound	RP
DF101	13	Bilateral profound congenital	RP
DF5	16	Bilateral profound congenital (90 dB)	RP, psychomotor delay
DF255	28	Bilateral profound congenital	RP
DF522	ND	Bilateral profound congenital	RP
DF618	ND	ND	RP
DF619	ND	ND	RP

Patients were classified in descending order according to age; RP: *retinitis pigmentosa*; ND: not determined.

Among the 10 investigated cases, audiological examinations revealed bilateral profound deafness in eight patients. A familial history of HI has been reported in five consanguineous families.

Molecular investigation by TGS in the 10 USH patients allowed the identification of 10 different variants in 5 genes, namely *MYO7A* (*n* = 6), *USH1G* (*n* = 1), *PCDH15* (*n* = 1), *CDH23* (*n* = 1), and *USH2A* (*n* = 1), which highlights the genetic and mutational heterogeneity of this syndrome even in a clinically homogeneous group of patients (9 USH1 cases) (Table 2). These variants included a duplication and an insertion that lead to a frameshift, three variants affecting splice sites, a missense variant, and a large deletion. The genetic diagnosis was confirmed by ophthalmologic fundus examination using ERG and OCT.

**TABLE 2:** Molecular findings in patients with Usher syndrome.

Patient code	Age (years)	Geographical location	Consanguinity	Gene	Nucleotide change	Amino-acid change	Zygosity	Location
DF5	16	Northern Tunisia	N	*USH1G*	c.84dup	p.(Asp29Glyfs29)	HOM	Exon 1
DF101	13	Southern Tunisia	Y	*PCDH15*	c.4543_4544insA	p.(Pro1515Hisfs4)	HOM	Exon 39
DF159	11	Northern Tunisia	Y	*MYO7A*	c.6487G>A	p.(Gly2163Ser)	HOM	Exon 48
DF202	9	Northern Tunisia	Y	*CDH23*	c.2953 + 1G>A	NA	HOM	Intron 25
DF211	9	Southern Tunisia	Y	*MYO7A*	Deletion of exon 4	ND	HOM	Exon 4
DF252	8	Central Tunisia	Y	*MYO7A*	c.2283-1G>T	NA	HOM	Intron 19
DF255	28	Northern Tunisia	Y	*MYO7A*	c.3285 + 1G>T	NA	HOM	Intron 25
DF522	ND	Northern Tunisia	Y	*MYO7A*	c.5434G>A	p.(Glu1812Lys)	HOM	Exon 39
DF619	ND	ND	ND	*MYO7A*	c.5581C>T	p.(Arg1861)	HOM	Exon 40
DF618	ND	ND	ND	*USH2A*	c.5776 + 1G>A	NA	HOM	Intron 28

Variant identifier in dbSNP	Variant type	Clinical significance in ClinVar	Pathogenicity classification criteria according to ACMG	Inheritance pattern	Sequencing method	Clinical sub-type	References
rs751695203	Duplication	NR	Pathogenic: PVS1, PM2, PP3	AR	TGS	USH/I	Bonnet et al. (2011)
NR	Insertion	NR	Likely Pathogenic: PVS1, PM2	AR	TGS	USH/I	Present study
rs747656448	Missense	Pathogenic	Pathogenic: PP5, PM2, PM5, PP3	AR	TGS	USH/I	Janecke et al. (1999)
NR	Splicing	NR	Pathogenic: PVS1, PM2, PP3	AR	TGS	USH/I	Present study
NR	Large deletion	NR	Likely pathogenic: PVS1, PM2	AR	TGS	USH/I	Present study
rs397516295	Splicing	Pathogenic	Pathogenic: PVS1, PM2, PP3, PP5	AR	TGS	USH/I	Roux et al. (2006)
NR	Splicing	NR	Pathogenic: PVS1, PM2, PP3	AR	TGS	USH/I	Present study
rs377267777	Missense	Pathogenic/likely Pathogenic	Pathogenic: PP5, PM2, PP3	AR	TGS	USH/I	Roux et al. (2011)
rs878864531	Nonsense	Pathogenic	Pathogenic: PVS1, PM2, PP3, PP5	AR	TGS	USH/I	Adato et al. (1997)
rs876657731	Splicing	Pathogenic	Pathogenic: PVS1, PM2, PP3, PP5	AR	TGS	USH/II	Dreyer et al. (2008)

Patients were classified according to the clinical sub-type; NA: non applicable; ND: not determined; NR: not reported; HOM: homozygous; Y=Yes; N=No.

In patient DF211, no causative mutation was initially identified. Following the analysis of the quality control files that provide information on the coverage of the targeted exons in the gene panel, a lack of coverage (0 Reads in 1X, 4X, 10X, and 25X read depths) was detected in the genomic region covering exon 4 of the *MYO7A* gene. Therefore, we hypothesized the presence of a homozygous deletion in this particular genomic region. The CNV analysis conducted on TGS data using the R package ExomeDepth provided validation to our hypothesis, revealing a homozygous deletion encompassing exon 4 (151 bp) of the *MYO7A* gene. Following annotation by the AnnotSV tool, this CNV, leading to a loss of function, was classified as potentially pathogenic (class 4 according to the ACMG criteria). This deletion has not been reported in the DGV database or in the literature.

#### 3.2.2 Genetic investigation of Waardenburg syndrome

In the present study, 22 patients presenting with clinical features of Waardenburg syndrome (WS) and belonging to 16 unrelated families, were recruited. This is the largest WS Tunisian cohort reported to date since only six Tunisian patients have been described in the literature. Among the 16 families analyzed in the present work, a family history of isolated HI was reported in six families, three of which were multiplex for WS. The clinical diagnosis was made according to current international recommendations (Pingault et al., 2010; Trabelsi et al., 2017): the presence of at least two major clinical criteria or one major and two minor clinical criteria. Patients were also classified, according to the presence of additional clinical manifestations (Table 3). The W index was calculated to confirm the presence of dystopia canthorum (Trabelsi et al., 2017).

**TABLE 3:** Clinical characteristics of the patients presenting with Waardenburg syndrome.

Patient code	Age (years)	Hearing impairment degree	Associated clinical features
DF592-2	3	Severe bilateral	Blue irises, skin hypopigmentation
DF306	9	ND	Dystopia canthorum
DF290	11	ND	Sapphire-blue iris, gray hair, growth retardation
DF292	11	ND	Hirschsprung disease
DF289	13	ND	Heterochromia iridis, thoracic malformation, autistic features, hydrocephalus
DF285	14	ND	Heterochromia iridis, gait imbalance, obsessive behavioral disorder
DF40	15	Profound bilateral	Hirschsprung disease, heterochromia iridis, inner ear malformation: agenesis of lateral and posterior semicircular canals, vestibular dilatation
DF549-2	18	ND	Heterochromia iridis
DF549-1	20	ND	Heterochromia iridis
DF551-2	22	Severe bilateral	Heterochromia iridis, skin hypopigmentation
DF548	29	Profound bilateral	Polymalformative syndrome, hypertelorism, dystopia canthorum, narrowing of the nasal orifices, narrow nostrils, anti-mongoloid obliquity of the palpebral slits, hypoplasia of the lower maxillae
DF551-1	53	Severe bilateral	Heterochromia iridis, skin hypopigmentation
DF592-1	ND	Severe bilateral	Blue irises, skin hypopigmentation
DF41	ND	Profound bilateral	Hypertelorism, craniofacial dysmorphism, telecanthus

Patients were classified in descending order according to age; ND: not determined.

##### 3.2.2.1 Genetic investigation of WS cases with typical clinical presentations

The genetic investigation by TGS led to the identification of nine different variants in *SOX10 and PAX3* genes in nine patients from seven families (Table 4). Six variants, comprising 3 missense, 2 nonsense, and 1 splice acceptor site mutations were predicted as deleterious by at least six *in silico* prediction tools (Supplementary Table S1). The p.(Arg270Gly) missense variant in exon 5 of the *PAX3* gene as well as one inframe deletion (p.(Trp114del) and one nonsense variant p.(Trp114Ter)) in exon 2 of the *SOX10* gene were identified in three multiplex families (DF289, DF551, DF592). Given that the *SOX10* gene is involved in the molecular etiology of the five WS2 patients investigated by TGS, we performed a targeted mutational screening by Sanger sequencing of exon 2 of the *SOX10* gene in two cases belonging to the same family DF592-1 (mother) and DF592-2 (daughter) with WS2. This allowed the detection of a deleterious nonsense variant c.342G>A; p.(Trp114Ter) at the heterozygous state.

**TABLE 4:** Molecular findings in patients with Waardenburg syndrome.

Patient code	Age (years)	Geographical location	Consanguinity	Gene	Nucleotide change	Amino-acid change	Zygosity	Location
DF40	15	Northern-Tunisia	N	*SOX10*	c.698-1G>C	NR	HTZ	Intron3-exon4 junction
DF41	ND	Southern-Tunisia	N	*PAX3*	c.667C>T	p.(Arg223Ter)	HTZ	Exon 5
DF285	14	Northern-Tunisia	N	*SOX10*	c.356G>T	p.(Arg119Leu)	HTZ	Exon 2
DF289	13	Northern-Tunisia	N	*PAX3*	c.808C>G	p.(Arg270Gly)	HTZ	Exon 5
DF290	11	Southern-Tunisia	N	*SOX10*	c.385-386delCTinsGG	p.(Leu129Gly)	HTZ	Exon 2
DF292	11	Central-Tunisia	Y	*SOX10*	c.650del	p.(Pro217Glnfs69)	HTZ	Exon 3
DF551-1	53	Northern-Tunisia	N	*SOX10*	c.340_342del	p.(Trp114del)	HTZ	Exon 2
DF551-2	22	Northern-Tunisia	N	*SOX10*	c.340_342del	p.(Trp114del)	HTZ	Exon 2
DF592-1	ND	Northern-Tunisia	N	*SOX10*	c.342G>A	p.(Trp114Ter)	HTZ	Exon 2
DF592-2	3	Northern-Tunisia	ND	*SOX10*	c.342G>A	p.(Trp114Ter)	HTZ	Exon 2
DF306	9	Southern-Tunisia	N	*PAX3*	c.142G>T	p.(Gly48Cys)	HTZ	Exon 2

Variant identifier in dbSNP	Variant type	Clinical significance in ClinVar	Pathogenicity classification criteria according to ACMG	Inheritance pattern	Sequencing method	Clinical sub-type	References
NR	Splice acceptor site	NR	Pathogenic: PVS1, PM2, PP3	AD	TGS	WS/IV	Sznajer et al. (2008)
rs772241382	Nonsense	Pathogenic	Pathogenic: PVS1, PM2, PP3, PP5	AD	TGS	WS/III	Baldwin et al. (1995)
NR	Missense	NR	Likely pathogenic: PM2, PM1, PP2, PP3	AD	TGS	WS/II	Present study
NR	Missense	NR	Likely pathogenic: PM1, PM5, PM2, PP2, PP3	AD	TGS	WS/III	Niu et al. (2018)
NR	Indel	NR	Pathogenic: PM1, PM2, PM5, PP2,PP3	AD	TGS	WS/II	Present study
NR	Deletion	NR	Pathogenic: PVS1, PM2, PP3	AD	TGS	WS/IV	Present study
NR	Deletion	NR	Likely pathogenic: PM2, PM1, PM4, PP3	AD	TGS	WS/II	Present study
NR	Deletion	NR	Likely pathogenic: PM2, PM1, PM4, PP3	AD	TGS	WS/II	Present study
NR	Nonsense	NR	Pathogenic: PVS1, PM2, PP3	AD	Sanger sequencing	WS/II	Liu et al. (2020)
NR	Nonsense	NR	Pathogenic: PVS1, PM2, PP3	AD	Sanger sequencing	WS/II	Liu et al. (2020)
rs1419548558	Missense	Pathogenic/likely Pathogenic	Pathogenic: PM1, PM2, PM5, PP2, PP3, PP5	AD	Sanger sequencing	WS/I	Bocángel et al. (2018)

This table only includes molecular results obtained in patients with a clinically confirmed diagnosis of WS; patients were classified in descending order according to age; NR: not reported; ND: not determined; Y=Yes; N=N; WT, wild type.

##### 3.2.2.2 Diagnostic refinement: a case of craniofacial-deafness-hand syndrome (CFDH)

Patient DF548, aged 28 years and born to consanguineous parents, presented with a particular phenotype marked by the association of profound bilateral deafness with a dystopia canthorum and musculoskeletal malformations. Therefore, the diagnosis of WS3 was firstly considered. Since exons 2 and 5 of the *PAX3* gene have been reported as mutational hotspots in suspected WS1 and WS3 patients (Pingault et al., 2010), a targeted mutational screening of these 2 exons was performed in the proband. This led to the identification of a heterozygous missense variant p.(Asn47Lys) in exon 2 of the *PAX3* gene. This variant has been associated with CFDH which has been described in only three American familial cases carrying the same variant (Sommer et al., 1983).

This ultra-rare autosomal dominant syndrome is characterized by the association of profound deafness with dysmorphic features including a high forehead, flattened face, hypoplasia or absence of nasal bones, downwardly and outwardly directed palpebral slits, hypertelorism, dystopia canthorum, maxillary hypoplasia, and ulnar deviation of the hands (Asher et al., 1996; Sommer and Bartholomew, 2003). Since the clinical presentation of DF548 patient included hypertelorism, downwardly and outwardly directed palpebral slits, and maxillary hypoplasia, the clinical diagnosis has been refined to CFDH given that the aforementioned clinical signs are specific to CFDH rather than WS3.

To our knowledge, this is the second case of CFDH described in the world. Another missense variant p.(Asn47His) affecting codon 47 of the same gene and leading to the substitution of an asparagine by a histidine, has been previously associated with WS3 (Hoth et al., 1993). Two hypotheses have been proposed regarding the mechanisms involved in the variable expressivity of these two variants, namely, i) Different effects of the two variants on the affinity of the PAX-3 transcription factor to target genes and ii) Impact of modifier genes (Asher et al., 1996).

##### 3.2.2.3 Suspicion of a comorbidity associating deafness with iris heterochromia

In one sibling (DF549-1 and 2) with WS2 (HI associated with iris heterochromia), no mutations in WS-associated genes have been detected, however, a homozygous missense variant c.242G>A; p.(Arg81Gln) has been identified in exon 7 of the *LRTOMT* gene. This is a founder mutation frequently associated with non-syndromic HI (DFNB63) in North Africa (Tunisia, Morocco, Libya) (Ahmed et al., 2008; Charif et al., 2012; Mosrati et al., 2021; Souissi et al., 2021). To date, the *LRTOMT* gene has not been associated with a syndromic clinical entity. Such a result suggests the co-occurrence of the c.242G>A variant located in the *LRTOMT* gene, explaining the HI, with a second variant located in a yet unidentified gene involved in iris heterochromia.

#### 3.2.3 H syndrome (HS)

We report here, the clinical and genetic investigation of two Tunisian cases with H syndrome (HS) which is a genodermatosis first described in 2008 by Molho-Pessach et al. (Molho-Pessach et al., 2008). It belongs to a group of histiocytic diseases known as “Histiocytosis Lymphadenopathy Plus” (Morgan et al., 2010; Noavar et al., 2019). HS is an ultra-rare condition whose prevalence has been estimated to be less than one in 1,000,000 cases (Bhatti et al., 2018; El-Bassyouni et al., 2020). It is a multisystemic disorder characterized by hypoacusis, skin hyperpigmentation, hypertrichosis, heart disease, hypogonadism, hepatosplenomegaly, hyperglycemia, and hallux valgus.

The two patients, aged 21 (TUN_HS_8) and 32 (TUN_HS_10) years, were born to consanguineous parents originating from southern Tunisia. Both patients share several pathognomonic manifestations of HS, including hyperpigmentation, hypertrichosis, inflammatory syndrome, hallux valgus, bilateral camptodactyly of the toes, and heart anomaly. Other clinical signs were individually observed such as sacroiliitis, inflammatory joint arthralgias, chronic urticaria and hyperthyroidism in patient TUN_HS_08 as well as unilateral renal hypoplasia, hypogonadism, diabetes and hepatosplenomegaly in patient TUN_HS_10 (Table 5).

**TABLE 5:** Clinical characteristics of the patients presenting with H syndrome.

Patient code	Age (years)	Hearing impairment degree	Associated clinical features
TUN_HS_08	21	Moderate conductive (right ear); mild mixed (left ear)	Hyperpigmentation, hypertrichosis, chronic urticaria, dermis fibrosis, moderate interstitial and perivascular inflammatory infiltrate extended to the hypodermis composed of histiocytes, lymphocytes and plasma cells (CD68^+^), cardiac disease, hallux valgus, bilateral camptodactyly of the toes, osteoarticular abnormalities: unilateral right sacroiliitis of inflammatory origin, inflammatory arthralgias with arthritis of both wrists and left knee, hyperthyroidism
TUN_HS_10	32	Moderate congenital neurosensory bilateral	Hyperpigmentation, hypertrichosis, dermis fibrosis, cardiac disease: concentrically enlarged left ventricle, moderate pericardial effusion, hallux valgus, bilateral camptodactyly of the toes, ankylosing spondylitis associated with an osteoarticular disorder, moderate inflammatory syndrome, lympho-histiocytic infiltration (CD68^+^) of soft tissues, hepatosplenomegaly, diabetes mellitus, hypoplasia of right kidney, hypogonadism

The type and degree of HI was different between the two patients. TUN_HS_08 had an asymmetric bilateral HI; moderate conductive in the right ear and mild mixed in the left one, whereas TUN_HS_10 was diagnosed with a congenital bilateral moderate sensorineural HI.

The genetic etiology of HS was associated with compound heterozygous and homozygous variants in the *SLC29A3* gene encoding the balancing nucleoside transporter 3 (Hent3) located in endosomes, lysosomes, and mitochondria (Bhatti et al., 2018). Given that 36% (9/25 variants) of HS cases reported in the literature is related to variants located in exon 6 of the *SLC29A3* gene (Bolze et al., 2012; Al-Haggar et al., 2015), a targeted variant screening of this mutational hotspot was conducted by Sanger sequencing.

This revealed the presence of the missense variant c.1088G>A; p.(Arg363Gln) at the homozygous state in patient TUN_HS_08. In patient TUN_HS_10, missense variants p.(Arg363Gln) and c.971C>T; p.(Pro324Leu) were identified at the compound heterozygous state (Table 6). For both cases, parental segregation was not performed due to the unavailability of the parents’ DNA.

**TABLE 6:** Molecular findings in patients with H syndrome.

Patient code	Age (years)	Geographical location	Consanguinity	Gene (A)	Nucleotide change	Amino-acid change	Zygosity
TUN_HS_08	21	Southern-Tunisia	N	*SLC293*	c.1088G>A	p.(Arg363Gln)	HOM
TUN_HS_10	32	Southern-Tunisia	N	*SLC293*	c.971C>T/c.1088G>A	p.(Pro324Leu)/p.(Arg363Gln)	COMP HTZ

Location	Variant identifier in dbSNP	Variant type	Clinical significance in ClinVar	Pathogenicity classification criteria according to ACMG	Inheritance pattern	Sequencing method	References
Exon 6	rs387907066	Missense	Pathogenic	Likely pathogenic: PM2, PM5, PP5, PP3	AR	Sanger Sequencing	Molho-Pessach et al. (2010)
Exon 6	rs387907066/rs758201217	Missense	Pathogenic/Likely pathogenic	Likely pathogenic: PM2, PM5, PP5, PP3/Likely pathogenic: PM2, PP3, PP5	AR	Sanger Sequencing	Molho-Pessach et al. (2014). (Molho-Pessach et al. (2010)

N=No; HOM: homozygous; COMP HTZ: compound heterozygous.

### 3.3 Update of the spectrum of SDs in the Tunisian population

#### 3.3.1 Clinical specificities

Literature review conducted in the current study has allowed us to set up the phenotypic spectrum of SDs in the Tunisian population and to calculate the relative frequency of each syndrome (i.e., number of patients per clinical entity). It has allowed the identification of 231 patients affected with 31 different SDs, including relatively frequent syndromes as well as ultra-rare atypical clinical presentations. Among these SDs, four clinical entities correspond to comorbidities identified in four patients from four unrelated families.

**FIGURE 2:**
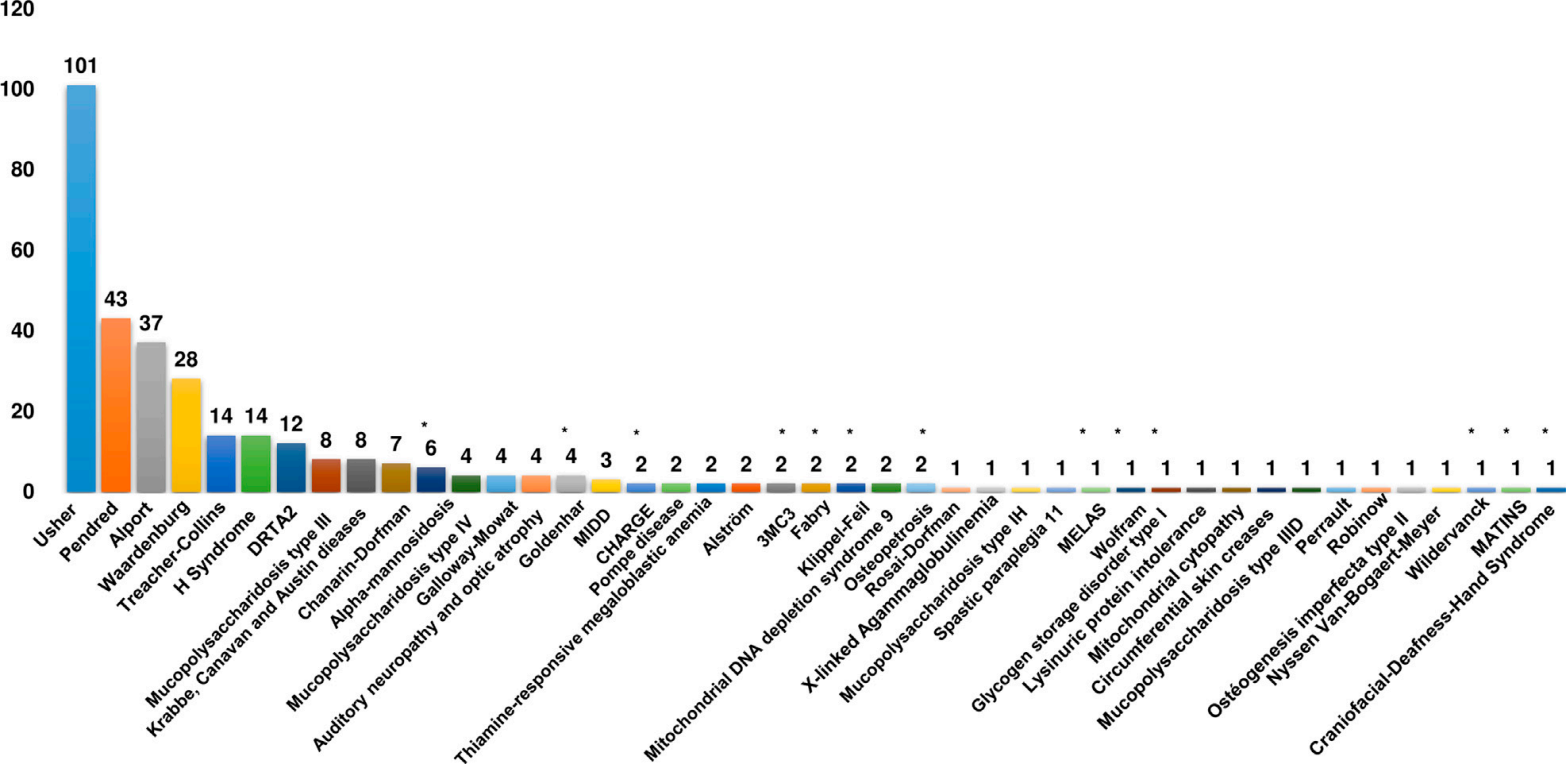
Clinical spectrum of syndromic deafness in the Tunisian population. Histograms recapitulating the number of patients for each syndromic deafness identified in Tunisia. The asterisk indicates syndromes that have only been reported in the current study. DRTA2: Renal tubular acidosis, distal, 2, with progressive sensorineural hearing loss; MIDD: Diabetes and deafness, maternally inherited.

On the other hand, the Patient census has led to expanding the phenotypic spectrum of SDs by identifying 11 syndromes not previously reported in the Tunisian population. By combining literature data with our results, we identified 309 Tunisian patients diagnosed with 42 different SDs. We also found that the top four SDs in Tunisia correspond to USH, PDS, AS, and WS with 101, 43, 37, and 28 cases, respectively (Figure 2).

Although it is an ultra-rare genodermatosis, HS with 14 identified patients is the sixth most common syndrome in Tunisia. The top four SDs are globally recognized as the most common ones with a slight difference regarding their ranks; for example, PDS is globally ranked as the most frequent SD followed by USH and WS (Koffler et al., 2015; Gettelfinger and Dahl, 2018). Among the listed SDs, 33% (14/42) fall into the category of syndromic non-specific forms.

SDs identified in Tunisia are mainly represented by two disease classes, i.e., congenital malformations, dysmorphic, and chromosomal aberrations (50%) as well as endocrine, nutritional, and metabolic diseases (48%).

Among the syndromic forms pertaining to the class of endocrine, nutritional, and metabolic diseases, 64% correspond to lysosomal storage disorders. The molecular basis of these dysfunctional conditions has been established in Tunisian patients affected with types IH, IIIB, and IIIC mucopolysaccharidosis, Pompe disease, and AM.

#### 3.3.2 Genetic specificities

##### 3.3.2.1 Classification of syndromic deafness according to the inheritance mode

**FIGURE 3:**
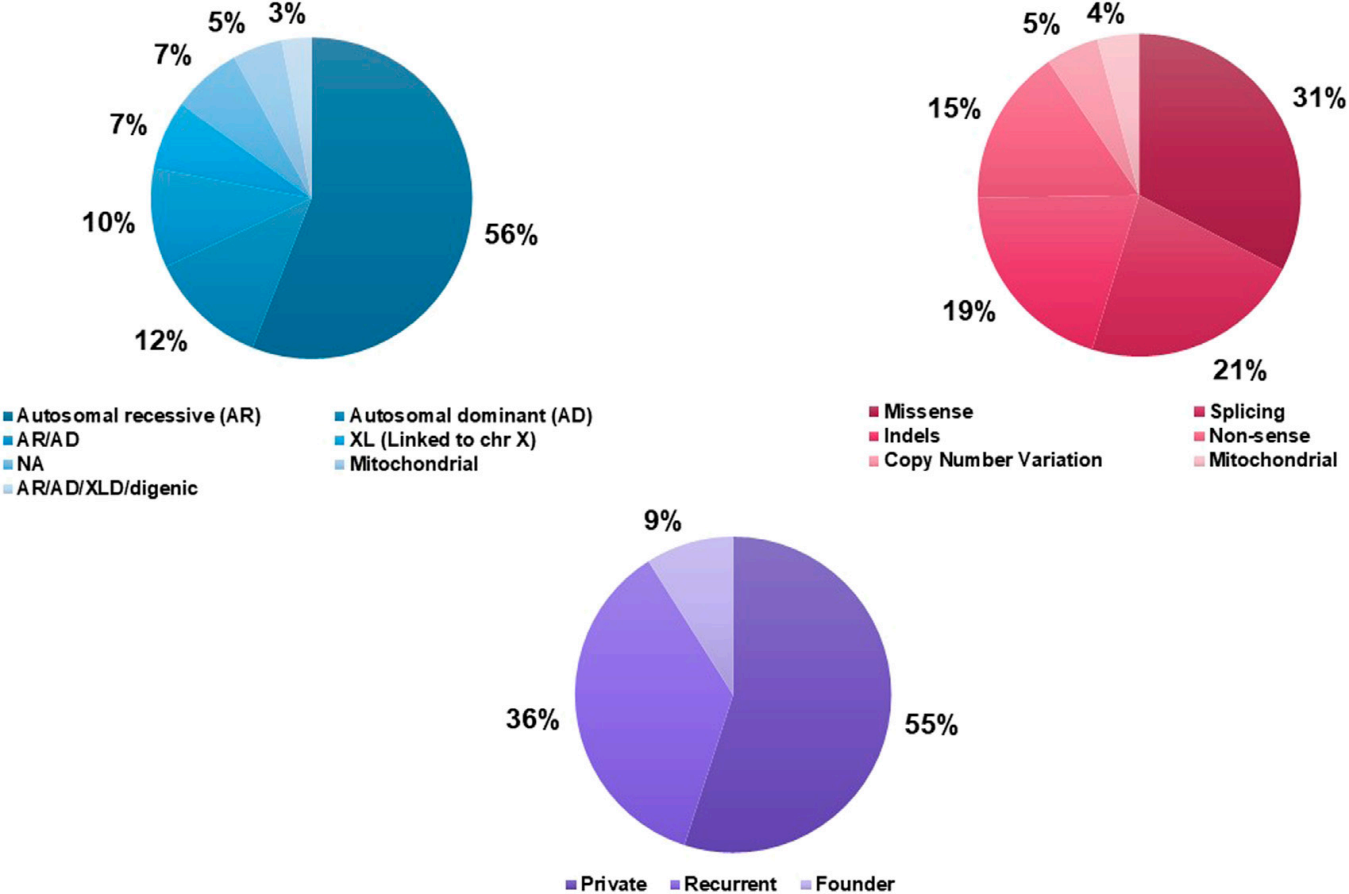
Genetic characteristics of Syndromic Deafness in the Tunisian population. **(A)** Inheritance patterns of syndromic deafness in Tunisia. **(B)** Causative variants types. **(C)** Recurrence of the causative variants.

In the investigated cohort (551 patients), the autosomal recessive mode of inheritance is the most frequent (34%) among the SDs identified. Those inherited in both recessive and dominant transmission modes accounted for 25% followed by strictly AD forms (17%). Inheritance patterns of 8% of the SDs detected in our cohort remain unknown due to the lack of molecular data. In total, when combining literature data with our findings, the rate of AR inheritance rises to 56% followed by the AD transmission mode (12%) (Figure 3A). Mitochondrial cytopathies represent 5% of all the SDs found in the Tunisian population.

##### 3.3.2.2 Genetic and mutational spectrum

According to published data, 50 causative variants in 23 nuclear and 3 mitochondrial genes were reported in 206 Tunisian patients with 19 different SDs. Variants detected in the different SDs were of 6 types; missense (31%), splicing (21%), frameshift deletions/duplications (19%), nonsense (15%), CNVs (5%), and mitochondrial (4%) (Figure 3B). Private variants represent the largest proportion (55%) followed by recurrent (36%), and founder (9.4%) mutations (Figure 3C, Supplementary Table S2).

Two founder mutations associated with the USH1B form (*MYO7A* gene) have been described in the Tunisian population. Two other founder mutations involved in non-specific SDs, namely, Chanarin-Dorfman syndrome and Mucopolysaccharidosis (MPS) type III, were specific to the Tunisian population (Supplementary Table S2).

The current study has broadened the genetic and mutational spectrum of genetic deafness by including 12 not previously reported variants in Tunisia and 9 others never described worldwide. These 21 variants were identified in 8 genes involved in 4 SDs, i.e., USH, WS, CFDH, and AM, that have been clinically confirmed in patients carrying these variants. For SW, SH, and AM, mutational hotspots were identified in *SOX10, SLC29A3, and MAN2B1* genes, respectively. Six variants not previously reported in Tunisia were identified in *CDH23 and SLC26A4* genes, associated with USH and PDS, respectively. Accordingly, in these patients, family segregation analysis of these two variants, combined with a thorough clinical evaluation will be performed to confirm their pathogenicity. Overall, the molecular data provided by the present study represent approximately 30% of all variants (22/72) identified in Tunisia.

**FIGURE 4:**
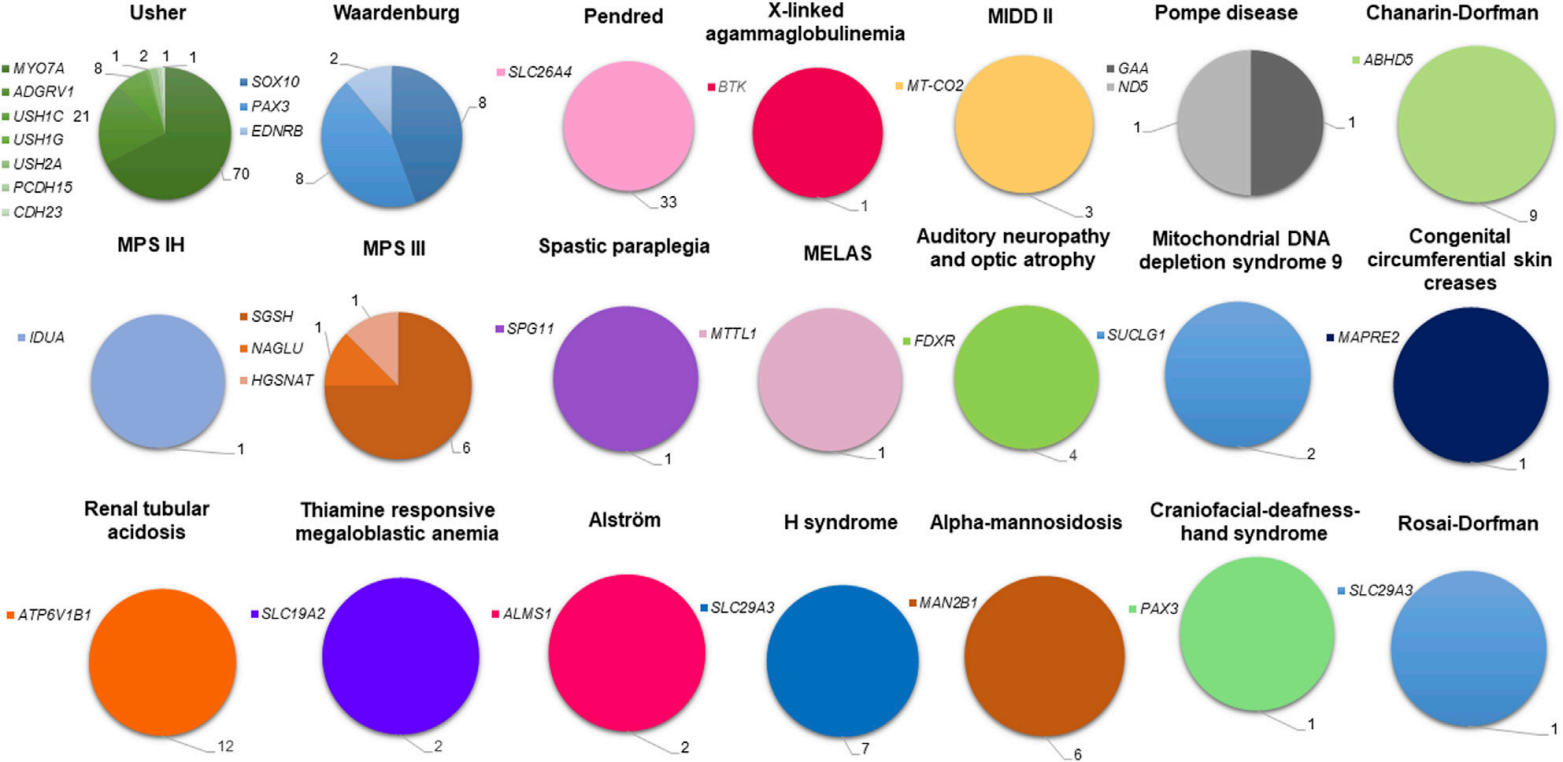
Genetic spectrum of syndromic deafness in the Tunisian population. Deafness syndromes with known molecular etiologies are represented by pie charts. Each syndromic deafness is indicated with a different color. Color degradation has been adopted to specify the gene(s) associated with each syndrome. The number of patients carrying variants in each gene is provided.

By merging our molecular findings with bibliography data, the proportion of cases with known molecular etiology in the Tunisian population is roughly 70% (234/334). The SDs diagnosed in the Tunisian population are marked by a high genetic heterogeneity which is far more important for USH whose molecular etiologies have been associated with 7 different genes (Figure 4).

## 4 Discussion

To the best of our knowledge, our study is the first to identify the spectrum of SDs in the Tunisian population. Tunisia is part of the lower medium income countries (LMIC; category 2; https://data.worldbank.org/country/TN). Its socio-economic, geographical position, and history of settlement, places the country at the crossroads of several regions including North-Africa, South-Europe and Middle East. Consequently, specific lessons learnt from Tunisia would aid in setting-up tailored clinical and genetic diagnosis approaches, thereby enhancing intervention and disease management. These insights may hold relevance beyond Tunisia and could potentially be replicated in other countries with shared genetic backgrounds.

Interestingly, our study reveals that syndromic deafness is encountered in major disease groups not necessarily classified as SD, such as genodermatosis, Chanarin-Dorfman syndrome, HS, Rosai-Dorfman syndrome, as well as hereditary metabolic disorders. These latter contribute to 33% (14/42) of the overall spectrum. Among these, Lysosomal storage diseases (Mucopolysaccharidosis, Pompe disease (PD), glycogen storage disease type II and AM) represent 64%.

One of the strengths of our study is the diversity of its data sources considering that our patient census, carried out in different medical specialty departments, was complemented by a literature survey. On the other hand, we have also identified the study limitations which mainly include the recruitment bias due to the focus of clinical specialists on certain conditions rather than others, such as the large number of Alport syndrome cases identified in the Congenital and Hereditary Diseases Department, considered as a national referral center for the genetic diagnosis of renal diseases. One other limitation is related to the estimates provided by the literature review, which may be skewed by data redundancy especially in the absence of a national registry for genetic HI in Tunisia. Nevertheless, the overall statistics would not deviate significantly since they would be rebalanced by the proportion of SDs that are still largely under-diagnosed. Although the spectrum of SDs here identified remains incomplete, it provides a substantial insight into the clinical and genetic specificities of SDs in the Tunisian population and reveals the significant proportion of hereditary metabolic disorders in this spectrum.

The classification of SDs is challenging due to several layers of complexity. These especially include the clinical heterogeneity with variable expressivity and disease outcome, resulting in a paucity of knowledge about SDs in the clinical practice. In terms of genetic characterization, SD classification is complex given the diversity of inheritance patterns, the genetic and mutational heterogeneity, the genetic background of the study population, as well as the socio-cultural context favoring consanguineous and endogamous marriages. Another layer of complexity is added by the fact that the healthcare system is characterized by a lack of adequate infrastructure and limited human and financial resources to ensure a thorough clinical and para-clinical investigation. For all these reasons, the risk of diagnostic error and delay in patient care is significant. Thus, genetic study remains the most reliable approach to obtain a precise diagnosis, to offer genetic counseling to families and to propose a prenatal diagnosis especially when the risk of recurrence is high.

Given the wide variety of SDs to consider, accurate clinical diagnosis could be challenging, even for an experienced clinician. Diagnostic errancy can be induced by strict phenotype-driven diagnostic methods, for this reason, some authors have suggested that the genomic approach should be given priority over the clinical approach (Monies et al., 2019). However, to take into account the unique clinical circumstances of each patient, it would be necessary to practice both approaches simultaneously in order to boost early diagnosis and to provide personalized medical management.

Historically, the genetic investigation of HI has been initiated in Tunisia since 1995, 1 year after the discovery of the first locus associated with ARNSHL (DFNB1) by Guilford et al. in two Tunisian inbred families (Guilford et al., 1994). Literature review has revealed that in 86% of the cases, genetic investigations of SDs were based on standard techniques such as linkage analysis, direct sequencing, and genotyping microarrays. Such approaches were mainly used for the genetic screening of recurrent and founder mutations for WS, PDS, HS, AM, and mitochondrial DNA depletion syndrome (Supplementary Table S2).

Twenty-five causative variants have been identified, in a 6-year time frame (2015–2021), *versus* 23 variants over an 18-year period (1995–2013) during the pre-NGS era (Supplementary Figure S1). This means that on average, 4 mutations per year were detected by NGS while only one variant per year was identified by conventional techniques. Thus, the detection rate has four-fold increase in less than a third of the time following the use of NGS techniques whose contribution to the genetic and mutational spectrum of SDs in Tunisia is equivalent to 37.5% (27/72 variants).

In the present work, the contribution of TGS to the detection rate is 2 times higher than the one obtained by Sanger sequencing (59.33% for TGS *versus* 29.25% for Sanger sequencing) (Supplementary Figure S2). TGS has also allowed to enrich the mutational spectrum of SDs by the identification of 21 novel variants not previously described in Tunisia (Tables 2 and 4). These results highlight the significant contribution of NGS to the molecular characterization of SDs in Tunisia.

Given the genetic and mutational heterogeneity of numerous SDs identified in Tunisia, a thorough genetic investigation based on Sanger sequencing would be impractical in time and cost-effectiveness. TGS has been consistently proven to be an effective approach for the genetic diagnosis of deafness with detection rates ranging between 16% and 42% (Sheppard et al., 2018). A panel of 120 genes has detected molecular etiologies in 33.5% of the investigated cases (N = 200) (Zazo Seco et al., 2017). The Otoscope gene panel has yielded a modest increase in diagnostic rate towards 39% (Sheppard et al., 2018). In the current study, the gene panel of choice resulted in a greater diagnostic yield, i.e. 59.33% (70 solved cases/118 investigated cases; Supplementary Figure S2) and allowed us to ascertain the molecular etiologies of USH and WS in 10 and 8 patients, respectively. However, it is important to note that all of the aforementioned gene panels were used for patients with apparently isolated HI.

Shearer et al. have emphasized the importance of including SD-associated genes in TGS panels to increase the detection rate of syndromic entities characterized by hidden clinical features such as RP in USH (Shearer et al., 2013; Sheppard et al., 2018). Indeed, this is well illustrated by the present study as our gene panel covering 46 SD genes allowed for the detection of USH-gene variants in five patients originally diagnosed with non-syndromic HI. Such findings suggest that the use of a specific gene panel for each SD would significantly increase the diagnostic yield. This was demonstrated in the study by Bonnet et al. who used an USH-specific gene panel that resulted in an 85% diagnostic yield (Bonnet et al., 2016). However, the use of syndrome-specific gene panels requires the availability of accurate clinical data, which is not always the case in Tunisia. This may be due to poor patient referral within the healthcare system as well as limited access to thorough ophthalmological examinations (OCT and ERG) allowing the detection of minor RP clinical signs. In the same study by Bonnet et al., TGS combined with CGH (Comparative Genome Hybridization) and qPCR (quantitative PCR) increased the detection rate of molecular etiology to 93% by identifying CNVs in patients carrying monoallelic point mutations in USH-associated genes (Bonnet et al., 2016). In two Tunisian familial cases, Adato et al. have reported a large deletion in the *MYO7A* gene, which was identified by linkage analysis and validated by Sanger sequencing (Adato et al., 1997). However, all these techniques (CGH, qPCR, Sanger) used for CNV identification and validation are highly expensive and time consuming. Indeed, our study showed the efficiency of a robust bioinformatics analysis, using the ExomeDepth tool, in the detection of CNVs from TGS data in the USH patient DF211. Therefore, we consider CNV analysis to be an essential step for negative TGS/WES cases affected with USH. We also propose long-range PCR as a cost-effective CNV validation technique when compared to qPCR.

The high rate of inbreeding characterizing the Tunisian population predicts an increasing emergence of rare and complex syndromes. Indeed, consanguinity and endogamy rates in Tunisia can reach 38% and 87%, respectively (Ben Halim et al., 2015). Among the 220 Tunisian SD families, including those reported in the literature, 66.81% of the index cases were born to consanguineous parents. This factor, in conjunction with the clinical and genetic heterogeneity of SD, could compromise the design of a gene panel adequately adapted to the genetic profile of the Tunisian population. In the present study, we found that the spectrum of SDs in Tunisia tends to constantly expand. Thus, the discovery of new syndromes, such as AM, will lead to an increase in the number of loci that need to be covered in a gene panel and thereby enhance its cost. As WES is increasingly becoming affordable, (Shearer et al., 2013), this tool would be more suitable for the genetic investigation of complex genetic conditions, namely, nonspecific and ultra-rare syndromic forms.

Given the limited access to NGS platforms in Tunisia, we propose as a first step, for the diagnosis of clinically confirmed SDs associated with a single gene, to proceed with direct sequencing of the mutational hot spots, e.g., AM and HS which are often associated with recurrent mutations. Furthermore, for familial cases, if the causal mutation segregating with the syndromic entity is known, cascade screening would be highly effective for the early detection of affected family members. For genetically heterogeneous forms that are clinically well characterized, we recommend the use of a gene panel, specific to the SDs frequently encountered in Tunisia such as USH, PDS, AS, and WS.

Our study showed that several SDs were originally classified as non-syndromic, which was the case for USH in the study cohort. Similarly, comorbidity cases were initially classified as SD, e.g., a co-occurrence of HI and ichthyosis that was primarily suspected as a KID syndrome in one patient (Sayeb et al., 2019). Such findings imply that each isolated deafness could be considered as a SD until proven otherwise. Likewise, each SD could actually be a comorbidity. In fact, for the syndromic forms for which deafness is the primary clinical manifestation, it is important to note that regular follow-up should be planned for all patients presenting with an apparently isolated deafness and carrying variants in the genes involved in USH or the *SLC26A4* gene associated with PDS in order to anticipate the diagnosis of RP or thyroid dysfunction for USH and PDS, respectively.

In light of our study, and considering the importance of the earliest possible intervention for children with congenital deafness, it is essential for these children to be detected as early as possible, at birth or during infancy, so that they can benefit from appropriate auditory and educational care.

Early detection of deafness through newborn hearing screening would allow in the mid to long term to implement a genetic screening system, especially in the presence of a family history and after excluding the involvement of non-genetic etiologies. Therefore, it is mandatory to combine clinical screening with genetic testing, if possible with NGS, in the newborn screening system. The failure to implement Universal Newborn Hearing Screening (UNHS) programs in Tunisia is due in part to a combination of several technical, financial, organizational and ethical considerations. To circumvent these limitations, knowledge societies and patient advocacy groups have become increasingly mindful towards the importance of awareness and advocacy campaigns to seek action from policymakers and stakeholders in Tunisia. Taking into account that the risk of developing a hearing impairment is inherent at all ages, all children should have in their health record a hearing health follow-up at crucial developmental stages: birth, preschool or school admission, and adolescence. The widespread use of the hearing health record should also be maintained throughout adulthood. This should be facilitated by the attribution of a unique identifier through the social security system and the implementation of the Electronic Medical Record (EMR). These two aspects have already been planned within the framework of the health policy and are being supported by the national dialogue on health in Tunisia.

In summary, the present study aims to raise awareness on the threats weighing on hearing health in the region, especially the genetic predisposition due to consanguinity and endogamy, which makes our population prone to an increased incidence of complex clinical entities. On the other hand, we emphasize on the importance of auditory rehabilitation (hearing aid and cochlear implant: CI) in terms of social inclusion and management of associated anomalies. Indeed, children with severe to profound HI can benefit greatly from early intervention via CI even if they suffer from other disabilities as it would not only provide auditory rehabilitation and speech improvement, but it would also enhance psychomotor and behavioral development. The need to provide auditory rehabilitation is even more critical for USH considering that early indication of CI would allow the patient to get optimal outcomes and gain the maximum capacity in auditory communication before the onset of visual loss. Furthermore, several studies have revealed the beneficial outcomes of CI particularly in HI due to genetic etiology. Thus, early auditory and genetic screening could yield better clinical outcomes. In fact, numerous studies have suggested that the CI clinical outcomes could be influenced by the genetic etiologies underlying HI. Good CI outcomes have been noted in patients affected with USH1 (Loundon et al., 2003), WS (Eshraghi et al., 2020; Polanski et al., 2020), Stickler, Bartter, Down, CINCA (Chronic Infantile Neurological Cutaneous and Articular), and Donnai-Barrow syndromes (Broomfield et al., 2013). However, outcomes can vary even in patients belonging to the same syndrome group. Such findings pinpoint the need to better understand genotype-phenotype correlations and CI outcome in order to provide effective genetic counseling and treatment strategies.

## Data Availability

The data presented in the study are deposited in the ClinVar repository under accession number SUB14263767.
