# Corrigendum: Current phenotypic and genetic spectrum of syndromic deafness in Tunisia: paving the way for precision auditory health

**DOI:** 10.3389/fgene.2024.1437233

**Published:** 2024-06-25

**Authors:** Rahma Mkaouar, Zied Riahi, Jihene Marrakchi, Nessrine Mezzi, Lilia Romdhane, Maroua Boujemaa, Hamza Dallali, Marwa Sayeb, Saida Lahbib, Hajer Jaouadi, Hela Boudabbous, Lotfi Zekri, Mariem Chargui, Olfa Messaoud, Meriem Elyounsi, Ichraf Kraoua, Anissa Zaouak, Ilhem Turki, Mourad Mokni, Sophie Boucher, Christine Petit, Fabrice Giraudet, Chiraz Mbarek, Ghazi Besbes, Soumeyya Halayem, Rim Zainine, Hamida Turki, Amel Tounsi, CRYSTEL Bonnet, Ridha Mrad, Sonia Abdelhak, Mediha Trabelsi, Cherine Charfeddine

**Affiliations:** ^1^ Laboratory of Biomedical Genomics and Oncogenetics LR16IPT05, Pasteur Institute in Tunis, University of Tunis El Manar, Tunis, Tunisia; ^2^ Department of Otorhinolaryngology, District Hospital of Menzel Bourguiba, Bizerte, Tunisia; ^3^ Department of Biology, Faculty of Sciences of Bizerte, Université Tunis Carthage, Tunis, Tunisia; ^4^ Genetic Typing Service, Institut Pasteur of Tunis, Tunis, Tunisia; ^5^ Marseille Medical Genetics (MMG) U1251, Aix Marseille Université, INSERM, Marseille, France; ^6^ Department of Pediatrics, La Rabta Hospital, Tunis, Tunisia; ^7^ Laboratory of Hereditary Diseases of the Metabolism Investigation and Patients Management, Faculty of Medicine in Tunis, University of Tunis El Manar, Tunis, Tunisia; ^8^ Department of Epidemiology and Public Health, Directorate General of Military Health, Faculty of Medicine in Tunis, University of Tunis El Manar, Tunis, Tunisia; ^9^ ICHARA Association (International Research Institute on Sign Language), Tunis, Tunisia; ^10^ Department of Congenital and Hereditary Diseases, Charles Nicolle Hospital in Tunis, Tunis, Tunisia; ^11^ LR99ES10 Laboratory of Human Genetics, Faculty of Medicine in Tunis, University of Tunis El Manar, Tunis, Tunisia; ^12^ Child and Adolescent Neurology Department of Neurology, National Institute of Neurology, Tunis, Tunisia; ^13^ LR18SP04 Department of Child Neurology, National Institute Mongi Ben Hmida of Neurology in Tunis. University of Tunis El Manar, Tunis, Tunisia; ^14^ Department of Dermatology, Habib Thameur Hospital, Research Unit Genodermatoses and Cancers LR12SP03, Tunis, Tunisia; ^15^ Service de dermatologie, Hôpital La Rabta, Unité de recherche UR 12SP07, Hôpital La Rabta, Tunis, Tunisia; ^16^ Service d’ORL et chirurgie cervico-faciale, CHU d’Angers, Angers, France; ^17^ Equipe Mitolab, Institut Mitovasc, CNRS UMR6015, UMR Inserm 1083, Université d’Angers, Angers, France; ^18^ Institut Pasteur, Université Paris Cité, Inserm UA06, Institut de l’Audition, Paris, France; ^19^ Collège de France, Paris, France; ^20^ Unité Mixte de Recherche (UMR) 1107, INSERM, Clermont-Ferrand, France; ^21^ Centre Auditif SoluSons, Clermont-Ferrand, France; ^22^ Service de Génétique Médicale, CHU de Clermont-Ferrand, Clermont-Ferrand, France; ^23^ ENT Department, Habib Thameur Hospital, Tunis, Tunisia; ^24^ Department of Otorhinolaryngology and Maxillofacial Surgery—La Rabta Hospital in Tunis, Tunis, Tunisia; ^25^ Service de pédopsychiatrie, Hôpital Razi, Faculté de Médecine de Tunis, Université Tunis el Manar, Tunis, Tunisia; ^26^ Dermatology Department Hedi Chaker University Hospital, Sfax University Sfax Tunisia, Tunis, Tunisia; ^27^ CNSS Polyclinic, Bizerte, Tunisia; ^28^ Université de la Manouba, Institut de Biotechnologie de Sidi–Thabet, Ariana, Tunisia

**Keywords:** syndromic deafness (SD), spectrum of SDs, next-generation sequencing (NGS), early detection, under-diagnosis, Tunisia

In the published article, there was an error in the legend for Figure 4 as published. The gene names corresponding to color degradation for the Waardenburg syndrome pie chart are incorrect.

**FIGURE 4:**
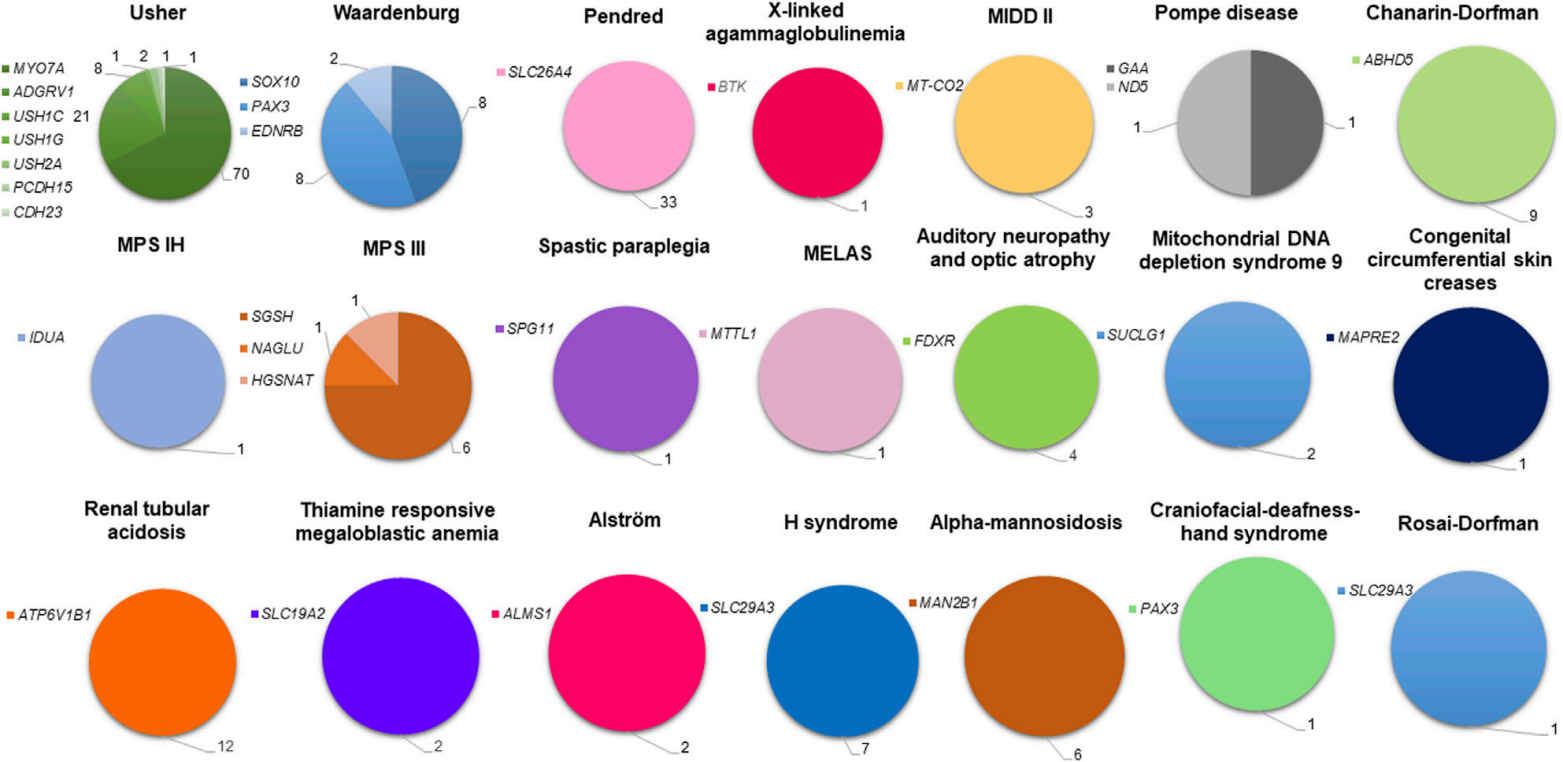
Genetic spectrum of syndromic deafness in the Tunisian population. Deafness syndromes with known molecular etiologies are represented by pie charts. Each syndromic deafness is indicated with a different color. Color degradation has been adopted to specify the gene(s) associated with each syndrome. The number of patients carrying variants in each gene is provided.

The corrected legend appears below: The SOX10 gene should be at the top (darkest blue), and the EDNRB gene should be at the bottom (lightest blue). Consequently, the number of patients should be 8 for the SOX10 gene and 2 for the EDNRB gene.

In addition, there was an error in the caption of Figure 4 in the published version of the manuscript: “FIGURE 4: Genetic and mutational spectrum of syndromic deafness in the Tunisian population. Deafness syndromes with known molecular etiologies are represented by pie charts. Each syndromic deafness is indicated with a different color. Color degradation has been adopted to specify the gene(s) associated with each syndrome. The number of variants identified in each gene is provided.”

The corrected Figure 4 appears below:

The authors apologize for these errors and state that this does not change the scientific conclusions of the article in any way. The original article has been updated.

