# Supplementary figures and images for: Current phenotypic and genetic spectrum of syndromic deafness in Tunisia: paving the way for precision auditory health
Source: Front Genet. 2024 Apr 22;15:1384094. doi: 10.3389/fgene.2024.1384094 (PMC11072975; doi:10.3389/fgene.2024.1384094)

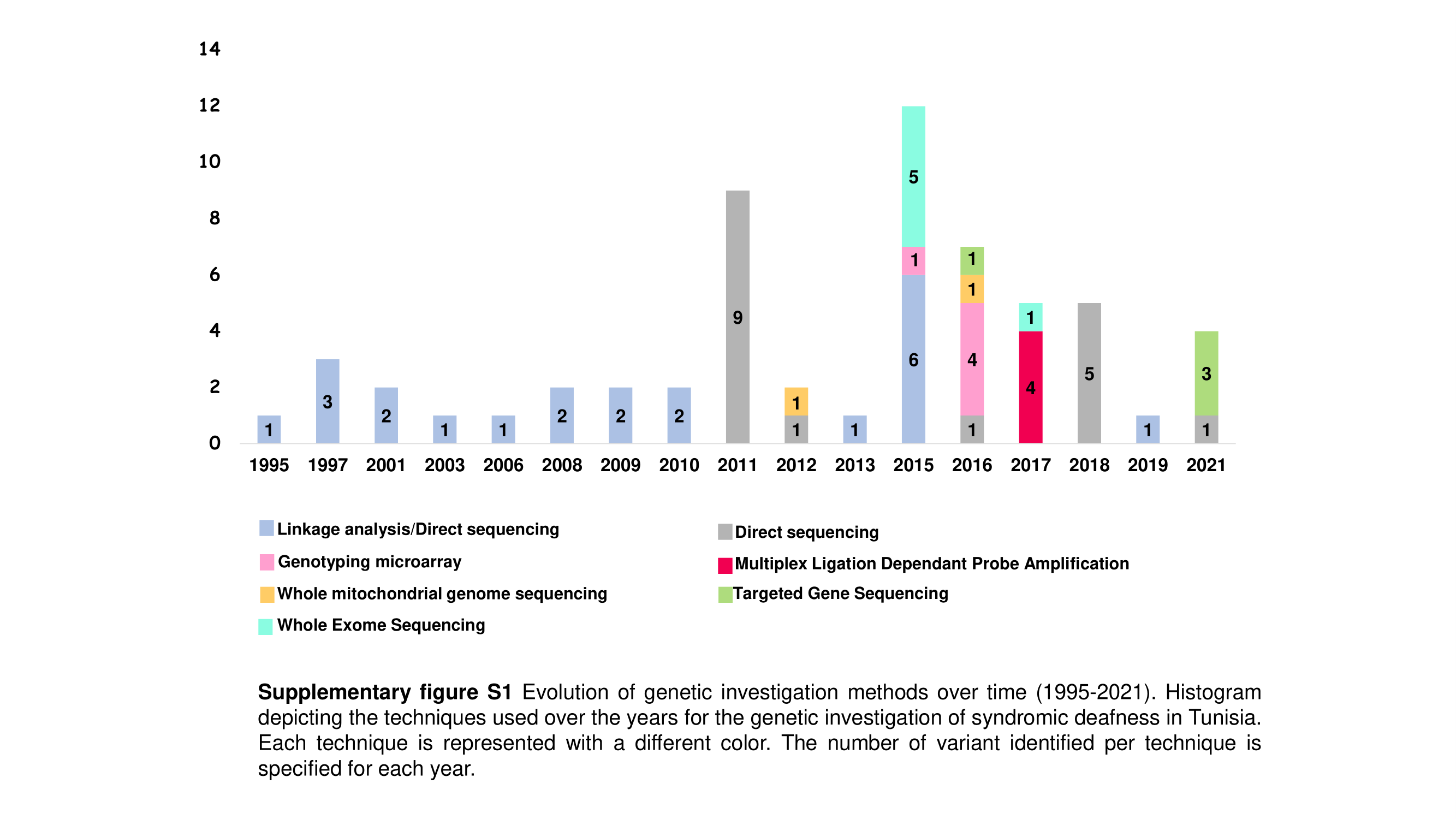

Supplement: Supplementary file 1 [file Image1.TIFF]

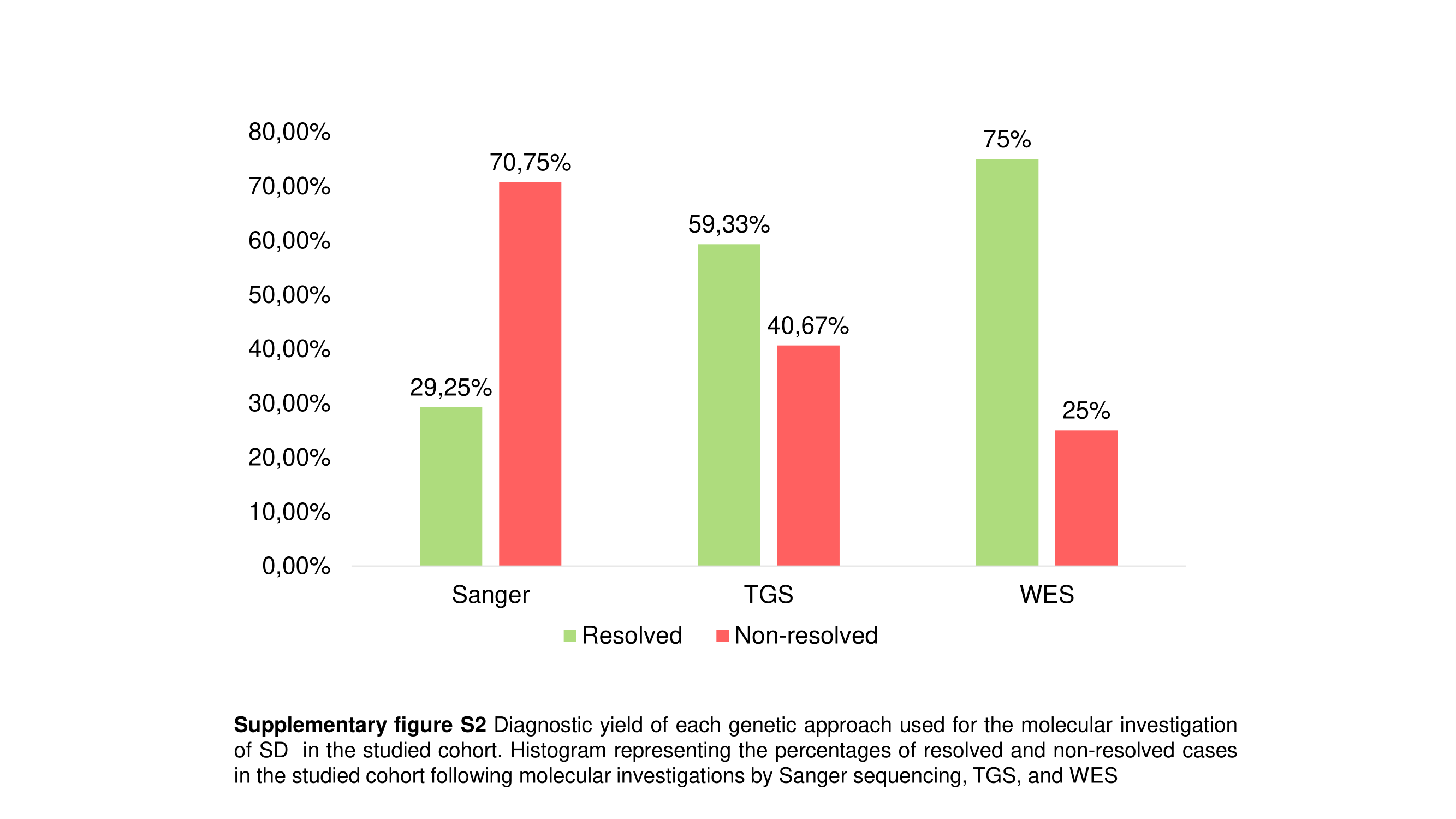

Supplement: Supplementary file 4 [file Image2.TIFF]
