# Supplementary material for: Current phenotypic and genetic spectrum of syndromic deafness in Tunisia: paving the way for precision auditory health
Source: Front Genet. 2024 Apr 22;15:1384094. doi: 10.3389/fgene.2024.1384094 (PMC11072975; doi:10.3389/fgene.2024.1384094)
Supplement: Supplementary file 2 [file Table1.DOCX]

**Table S1. In-silico prediction tool outcomes for the variants identified in Waardenburg syndrome patients**

| Gene | Nucleotide change | Amino-acid change | Inheritance pattern |  | Variant type | Clinical significance in ClinVar | Pathogenicity classification criteria according to ACMG | In-silico prediction tool outcomes | Reference |
| --- | --- | --- | --- | --- | --- | --- | --- | --- | --- |
| *SOX10* | c.698-1G>C | NR | AD |  | Splice acceptor site | NR | Pathogenic : PVS1, PM2, PP3 | dbscSNV, EIGEN, MaxEntScan, BayesDel addAF, BayesDel noAF: pathogenic strong; FATHMM-MKL: Damaging; MutationTaster: Disease causing | (Sznajer et al. 2008) |
| *PAX3* | c.667C>T | p.(Arg223Ter) | AD |  | Nonsense | Pathogenic | Pathogenic : PVS1, PM2, PP3, PP5 | FATHMM-MKL: Damaging; LRT: Deleterious; MutationTaster: Disease causing; BayesDel addAF, BayesDel noAF: pathogenic strong | (Baldwin et al. 1995) |
| *SOX10* | c.356G>T | p.(Arg119Leu) | AD |  | Missense | NR | Likely pathogenic : PM2, PM1, PP2, PP3 | MutPred, DEOGEN2, REVEL, MetaRNN: Pathogenic Strong; FATHMM, SIFT, LRT: Pathogenic Supporting | Present study |
| *PAX3* | c.808C>G | p.(Arg270Gly) | AD |  | Missense | NR | Likely pathogenic : PM1, PM5, PM2, PP2, PP3 | SIFT: deleterious; PolyPhen: probably_damaging; BayesDel addAF, BayesDel noAF, DEOGEN2: pathogenic strong; FATHMM-MKL: Damaging | (Niu et al. 2018) |
| *SOX10* | c.385-386delCTinsGG | p.(Leu129Gly) | AD |  | Indel | NR | Pathogenic : PM1, PM2, PM5, PP2,PP3 | SIFT: deleterious; Polyphen: probably_damaging | Present study |
| *SOX10* | c.650del | p.(Pro217Glnfs*69) | AD |  | Deletion | NR | Pathogenic : PVS1, PM2, PP3 | - | Present study |
| *SOX10* | c.340_342del | p.(Trp114del) | AD |  | Deletion | NR | Likely pathogenic : PM2, PM1, PM4, PP3 | - | Present study |
| *SOX10* | c.342G>A | p.(Trp114Ter) | AD |  | Nonsense | NR | Pathogenic : PVS1, PM2, PP3 | MutationTaster: Disease causing; LRT: Deleterious; EIGEN: Pathogenic Strong; BayesDel addAF, BayesDel noAF: Pathogenic Strong; FATHMM-MKL: Damaging | (Liu et al. 2020) |
| *PAX3* | c.142G>T | p.(Gly48Cys) | AD |  | Missense | Pathogenic/likely Pathogenic | Pathogenic : PM1, PM2, PM5, PP2, PP3, PP5 | MutPred, MetaLR, MetaRNN, REVEL, BayesDel addAF, BayesDel noAF: Pathogenic Strong; FATHMM-MKL, LRT, SIFT: Damaging/Pathogenic Supporting | (Bocángel et al. 2018) |

NR: Not Reported; ND : Not Determined
