# Supplementary material for: Current phenotypic and genetic spectrum of syndromic deafness in Tunisia: paving the way for precision auditory health
Source: Front Genet. 2024 Apr 22;15:1384094. doi: 10.3389/fgene.2024.1384094 (PMC11072975; doi:10.3389/fgene.2024.1384094)
Supplement: Supplementary file 3 [file Table2.DOCX]

**Table S1. Variants associated with deafness syndromes reported in the literature**

| Syndrome/Disease | Gene | Molecular etiology  (Nucleotide change) | Variant type | Founder/recurrent/ private | Technique | Number of patients | Consanguinity | Geographic location | Clinical sub-type | Reference(s) |
| --- | --- | --- | --- | --- | --- | --- | --- | --- | --- | --- |
| Usher | *MYO7A* | **c.470+1G>A/c.470+1G>A** | Splice donor site | Founder | Haplotyping analysis, direct sequencing (Sanger) | 2^Ϭ^ | Y | NA | I | (1) |
| Usher | *MYO7A* | **c.470+1G>A/c.470+1G>A** | Splice donor site | Founder | Genotyping microarray (NADf) | 1 | NA | NA | I | (2) |
| Usher | *MYO7A* | **c.470+1G>A/c.470+1G>A** | Splice donor site | Founder | Linkage analysis, genotyping microarray (NADf), direct sequencing (Sanger) | 24**^ϯ^** | N | Southern-Tunisia | I | (3) |
| Usher | *MYO7A* | **c.470+1G>A/c.2283-1G>T** | Splice donor site/Splice acceptor site | Founder/Recurrent | Targeted Gene Sequencing | 1 | N | NA | I | (4) |
| Usher | *MYO7A* | c.2119–2215del2kb/c.2119–2215del2kb | Large Deletion | Private | Haplotyping analysis, direct sequencing (Sanger) | 2 ^Ϭ^ | N | NA | I | (1) |
| Usher | *MYO7A* | c.1797G>A/c.1797G>A | Splicing | Private | Direct sequencing (Sanger) | 22 | Y | NA | I | (5) |
| Usher | *ADGRV1* | c.18131A>G/c.18131A>G | Missense | Private | Genotyping, Direct sequencing (Sanger) | 21^Ϭ^ | Y | NA | II | (6) |
| Usher | *MYO7A* | **c.1935G>A/ c.1935G>A** | Missense | Founder | Genotyping, Direct sequencing (Sanger) | 2 | N | Southern-Tunisia | I | (7) |
| Usher | *MYO7A* | **c.1935G>A/ c.1935G>A** | Missense | Founder | Genotyping, Direct sequencing (Sanger) | 1 |  | Southern-Tunisia | I | (2) |
| Usher | *MYO7A* | c.491A>G/c.491A>G | Missense | Private | Haplotyping analysis, Direct sequencing (Sanger)b | 1 |  | NA | I | (2, 8) |
| Usher | *MYO7A* | c.3508G>A/c.3508G>A | Missense | Recurrent | Genotyping microarray (NADf) | 1 |  | NA | I | (2) |
| Usher | *MYO7A* | c.1679A>G/c.1679A>G | Missense | Private | Targeted Gene Sequencing | 1 | Y | NA | I | (4) |
| Usher | *USH1C* | c.91C>T/c.91C>T | Nonsense | Recurrent | Genotyping microarray (NADf) | 1 |  | NA | I | (2) |
| Usher | *USH1C* | c.360C>T/c.360C>T | Missense | Private | Targeted Gene Sequencing | 1 | Y | NA | I | (4) |
| Usher | *USH1G* | **c.393insG/c.393insG** | Insertion (*Frameshift*) | Recurrent | Linkage analysis, direct sequencing | 8 ^Ϭ^ | N | Southern-Tunisia | I | (9) |
| Usher | *USH1G* | **c.393insG/c.393insG** | Insertion (*Frameshift*) | Recurrent | Genotyping microarray (NADf) | 1 | NA | Southern-Tunisia | I | (2) |
| Usher | *USH2A* | c.2212delA/c.2212delA | Nonsense | Private | Genotyping microarray (NADf) | 1 |  | NA | II | (2) |
| Usher | *MYO7A* | **c.2283-1G>T/c.2283-1G>T** | Splice acceptor site | Recurrent | Whole Exome Sequencing | 1 | Y (3/4 cases) | Central-Tunisia | I | (10) |
| Usher | *MYO7A* | c.5434G>A/c.5434G>A | Missense | Recurrent | Whole Exome Sequencing | 1 | Y (3/4 cases) | Northern-Tunisia | I | (10) |
| Usher | *USH1G* | c.1195_1196delAG/ c.1195_1196delAG | Deletion (*Frameshift*) | Private | Whole Exome Sequencing | 1 | Y (3/4 cases) | Northern-Tunisia | I | (10) |
| Usher | *USH1G* | c.52A>T/c.52A>T | Nonsense | Private | Whole Exome Sequencing | 1 | Y (3/4 cases) | Northern-Tunisia | I | (10) |
| Usher | *MYO7A* | c.1845delG/c.1845delG | Deletion  (*Frameshift*) | Private | Linkage analysis, genotyping microarray (NADf), direct sequencing (Sanger) | 24 **^ϯ^** | Y | Southern-Tunisia | I | (3) |
| Usher | *USH1C* | c.7C>T/c.7C>T | Nonsense | Private | Linkage analysis, genotyping microarray (NADf), direct sequencing (Sanger) | 2 ^Ϭ^ | Y | Southern-Tunisia | I | (3) |
| Usher | *PCDH15* | c.400C>T/c.400C>T | Nonsense | Recurrent | Linkage analysis, genotyping microarray (NADf), direct sequencing (Sanger) | 2 ^Ϭ^ | Y | Southern-Tunisia | I | (3) |
| Waardenburg | *EDNRB* | c.818C>G/c.818C>G | Missense | Private | Direct sequencing (Sanger) | 2 ^Ϭ^ | Y | NA | IV | (11) |
| Waardenburg | *PAX3* | c.942delC/WT | Deletion (*Frameshift*) | Private | Multiplex Ligation-dependent Probe Amplification | 1 | NA | NA | I | (12) |
| Waardenburg | *PAX3* | c.933_936dupTTAC/WT | Duplication (*Frameshift*) | Private | Multiplex Ligation-dependent Probe Amplification | 1 | NA | NA | I | (12) |
| Waardenburg | *PAX3* | c.164delTCCGCCA/WT | Deletion | Private | Multiplex Ligation-dependent Probe Amplification | 1 | NA | NA | I | (12) |
| Waardenburg | *PAX3* | Exons 5, 6, 7, 8, 9 | Large Deletion | Private | Multiplex Ligation-dependent Probe Amplification | 1 | NA | NA | I | (12) |
| Pendred | *SLC26A4* | **c.1334T>G/c.1334T>G** | Missense | Founder | Genotyping microarray (NADf), direct sequencing (Sanger) | 14 (8 families) | Y | Southern-Tunisia | NA | (13) |
| Pendred | *SLC26A4* | **c.1334T>G/c.1334T>G** | Missense | Founder | Direct sequencing (Sanger) | 23 (3 families) | Y | Southern-Tunisia | NA | (14) |
| Pendred | *SLC26A4* | c.451delG/c.451delG | Deletion (*Frameshift*) | Private | Genotyping, direct sequencing (Sanger) | 6 ^Ϭ^ | Y | NA | NA | (15) |
| Alström | *ALMS1* | c.10388-2A>G/c.10388-2A>G | Splicing | Private | Linkage analysis, Targeted Gene Sequencing (Puce TaGSCAN) | 2 | N^*^ | NA | NA | (16) |
| MIDD  (*Maternally Inherited Diabetes And Deafness*) | *MT-CO2* | m.8241 T >G/m.8241 T >G | NA | Private | Whole mitochondrial genome sequencing | 3 ^Ϭ^ | Y | NA | NA | (17) |
| MELAS (*Mitochondrial Myopathy, Encephalopathy, Lactic Acidosis, And Stroke-Like Episodes*) | *MTTL1* | m.1640A> G/ m.1640A> G | NA | Private | Whole mitochondrial genome sequencing | 1 | N | NA | NA | (18) |
| Tubular renal acidosis type 2 with sensorineural progressive hearing loss | *ATP6V1B1* | IVS2-1 G>C/IVS2-1 G>C | Splice acceptor site | Founder | Linkage analysis, direct sequencing (Sanger) | 12 | Y (2/10 cases) | Southern and Central-Tunisia | NA | (19, 20) |
| Auditory neuropathy and optic atrophy (*Mitochondrial Fe-S-Synthesis disease*) | *FDXR* | c.916C>T/c.916C>T | Missense | Recurrent | Whole Exome Sequencing | 4 ^Ϭ^ | Y | NA | NA | (21) |
| Mitochondrial DNA depletion syndrome type 9 | *SUCLG1* | c.41T > C/c.41T > C | Missense | Recurrent | Direct sequencing (Sanger) | 2 ^Ϭ^ | Y | NA | Encephalomyopathy with methylmalonic aciduria | (22) |
| Chanarin-Dorfman | *ABHD5* | c.774-1G>A/c.774-1G>A | Splicing | Founder | Linkage analysis, haplotyping analysis, direct sequencing (Sanger) | 1  8 | NA  NA^*^ | Southern-Tunisia  Southern-Tunisia | NA  Associated with hypothyroidism | (23)  (24) |
| H Syndrome | *SLC29A3* | **c.1088G>A/c.1088G>A** | Missense | Recurrent | Direct sequencing (Sanger) | 1  1  1  2 | Y  Y  Y  Y | NA  Southern-Tunisia  NA  Central-Tunisia | NA  NA  NA  NA | (25)  (26)  (27)  (28) |
| H Syndrome | *SLC29A3* | **c.1088G>A**/c.300 + 1G > A | Missense/splicing | Recurrent/ND | Direct sequencing (Sanger) | 1 | NA | NA | NA | (29) |
| H Syndrome | *SLC29A3* | c.971C>T/c.971C>T | Missense | Recurrent | Direct sequencing (Sanger) | 2 | Y | Southern-Tunisia | NA | (26) |
| H Syndrome | *SLC29A3* | c.42delC/c.42delC | Deletion  (*Frameshift*) | Private | Direct sequencing (Sanger) | 1 | Y | Southern-Tunisia | NA | (26) |
| Rosai-Dorfman | *SLC29A3* | **c.1088G>A/c.1088G>A** | Missense | Recurrent | Direct sequencing (Sanger) | 1 | Y | Central-Tunisia | NA | (28) |
| Symmetric circumferential skin creases, congenital, 2 | *MAPRE2* | c.260A>G/+ | Missense | Private | Whole Exome Sequencing | 1 | Y | NA | 2 | (30) |
| Pompe disease (Glycogenosis type II) | *GAA*  *ND5* | c.236_246del/c.236_246del  m.12908T>A/m.12908T>A | Deletion  Missense | Private  Private | Direct sequencing (Sanger), whole mitochondrial genome sequencing | 1 | Y | NA | II | (31) |
| Mucopolysaccharidosis type IH (Hurler Syndrome) | *IDUA* | c.1598C>G/c.1598C>G | Missense | Founder | Direct sequencing (Sanger) | 1 | Y | Southern-Tunisia | I | (32) |
| Mucopolysaccharidosis type III (San Filipo Syndrome) | *SGSH* | c.2T>C/c.2T>C | *Start loss* | Private | Direct sequencing (Sanger) | 1 | Y | Southern-Tunisia | IIIA | (33, 34) |
| Mucopolysaccharidosis type III | *SGSH* | c.1129C>T/**g.75802301_75809393del** | Missense/Deletion | Recurrent/Private | Direct sequencing (Quantitative Multiplex PCR of Short Fluorescent Fragments) | 1 | N | Central-Tunisia | IIIA | (33, 34) |
| Mucopolysaccharidosis type III | *SGSH* | c.1093C>T/c.1093C>T | Nonsense | Recurrent | Direct sequencing (Sanger) | 1 | Y | Northern-Tunisia | IIIA | (33, 34) |
| Mucopolysaccharidosis type III | *SGSH* | c.29dup/c.29dup | Duplication  (*Frameshift*) | Private | Direct sequencing (Sanger) | 1 | Y | Northern-Tunisia | IIIA | (33, 34) |
| Mucopolysaccharidosis type III | *SGSH* | c.1080del/c.1080del | Deletion  (*Frameshift*) | Private | Direct sequencing (Sanger) | 1 | Y | Northern-Tunisia | IIIA | (33, 34) |
| Mucopolysaccharidosis type III | *SGSH* | **g.75802301_75809393del**/ **g.75802301_75809393del** | Deletion | Private | Direct sequencing (Quantitative Multiplex PCR of Short Fluorescent Fragments) | 1 | Y | Central-Tunisia | IIIA | (33, 34) |
| Mucopolysaccharidosis type III | *NAGLU* | c.1674C>G/c.1674C>G | Nonsense | Private | Direct sequencing (Sanger) | 1 | Y | Central-Tunisia | IIIB | (33, 34) |
| Mucopolysaccharidosis type III | *HGSNAT* | c.1209G>A/c.1880A>G | Nonsense/missense | Founder/founder | Direct sequencing (Sanger) | 1 | Y | Central-Tunisia | IIIC | (33, 34) |
| X-linked agammaglobulinemia | *BTK* | c.1631 +1G >A/c.1631 +1G >A | Splicing | Private | Direct sequencing (Sanger) | 1 | NA | NA | XLA | (35) |
| Thiamine-responsive megaloblastic anemia | *SLC19A2* | c.287delG/c.287delG | Deletion  (*Frameshift*) | Private | Haplotyping analysis, direct sequencing (Sanger) | 2 ^Ϭ^ | Y | NA | NA | (36) |
| Spastic paraplegia 11 | *SPG11* | c.6100C>T/c.6100C>T | Nonsense | Recurrent | Linkage analysis, direct sequencing (Sanger) | 1 | NA | Southern-Tunisia | NA | (37, 38) |

Variants marked in bold were reported in more than one article; Ϭ: this symbol indicates cases belonging to a single family; ϯ: The 28 patients belong to 4 families but the number of cases per family was not specified in the corresponding article; *: Endogamous family; NA: Not Available; ND: Not Determined

1. Adato A, Weil D, Kalinski H, Pel-Or Y, Ayadi H, Petit C, et al. Mutation profile of all 49 exons of the human myosin VIIA gene, and haplotype analysis, in Usher 1B families from diverse origins. The American Journal of Human Genetics. 1997;61(4):813-21.

2. Chakchouk I, Ben Said M, Jbeli F, Benmarzoug R, Loukil S, Smeti I, et al. NADf chip, a two-color microarray for simultaneous screening of multigene mutations associated with hearing impairment in North African Mediterranean countries. J Mol Diagn. 2015;17(2):155-61.

3. Ben-Rebeh I, Grati M, Bonnet C, Bouassida W, Hadjamor I, Ayadi H, et al. Genetic analysis of Tunisian families with Usher syndrome type 1: toward improving early molecular diagnosis. Molecular vision. 2016;22:827.

4. Souissi A, Gibriel AA, Masmoudi S. Genetics and meta-analysis of recessive non-syndromic hearing impairment and Usher syndrome in Maghreb population: lessons from the past, contemporary actualities and future challenges. Human Genetics. 2021:1-11.

5. Weil D, Küssel P, Blanchard S, Lévy G, Levi-Acobas F, Drira M, et al. The autosomal recessive isolated deafness, DFNB2, and the Usher 1B syndrome are allelic defects of the myosin-VIIA gene. Nature genetics. 1997;16(2):191-3.

6. Hmani-Aifa M, Benzina Z, Zulfiqar F, Dhouib H, Shahzadi A, Ghorbel A, et al. Identification of two new mutations in the GPR98 and the PDE6B genes segregating in a Tunisian family. Eur J Hum Genet. 2009;17(4):474-82.

7. Rebeh IB, Morinière M, Ayadi L, Benzina Z, Charfedine I, Feki J, et al. Reinforcement of a minor alternative splicing event in MYO7A due to a missense mutation results in a mild form of retinopathy and deafness. Molecular vision. 2010;16:1898.

8. Roux A-F, Faugere V, Le Guedard S, Pallares-Ruiz N, Vielle A, Chambert S, et al. Survey of the frequency of USH1 gene mutations in a cohort of Usher patients shows the importance of cadherin 23 and protocadherin 15 genes and establishes a detection rate of above 90%. Journal of medical genetics. 2006;43(9):763-8.

9. Weil D, El-Amraoui A, Masmoudi S, Mustapha M, Kikkawa Y, Lainé S, et al. Usher syndrome type I G (USH1G) is caused by mutations in the gene encoding SANS, a protein that associates with the USH1C protein, harmonin. Hum Mol Genet. 2003;12(5):463-71.

10. Riahi Z, Bonnet C, Zainine R, Lahbib S, Bouyacoub Y, Bechraoui R, et al. Whole exome sequencing identifies mutations in Usher syndrome genes in profoundly deaf Tunisian patients. PLoS one. 2015;10(3):e0120584.

11. Attié T, Till M, Pelet A, Amiel J, Edery P, Boutrand L, et al. Mutation of the endothelin-receptor B gene in Waardenburg-Hirschsprung disease. Hum Mol Genet. 1995;4(12):2407-9.

12. Trabelsi M, Nouira M, Maazoul F, Kraoua L, Meddeb R, Ouertani I, et al. Novel PAX3 mutations causing Waardenburg syndrome type 1 in Tunisian patients. International Journal of Pediatric Otorhinolaryngology. 2017;103:14-9.

13. Rebeh IB, Yoshimi N, Hadj‐Kacem H, Yanohco S, Hammami B, Mnif M, et al. Two missense mutations in SLC26A4 gene: a molecular and functional study. Clinical genetics. 2010;78(1):74-80.

14. Charfeddine I, Mnejja M, Hammami B, Chakroun A, Masmoudi S, Ayadi H, et al. Pendred syndrome in Tunisia. Eur Ann Otorhinolaryngol Head Neck Dis. 2010;127(1):7-10.

15. Said MB, Dhouib H, BenZina Z, Ghorbel A, Moreno F, Masmoudi S, et al. Segregation of a new mutation in SLC26A4 and p. E47X mutation in GJB2 within a consanguineous Tunisian family affected with Pendred syndrome. International journal of pediatric otorhinolaryngology. 2012;76(6):832-6.

16. Chakroun A, Ben Said M, Ennouri A, Achour I, Mnif M, Abid M, et al. Long-term clinical follow-up and molecular testing for diagnosis of the first Tunisian family with Alström syndrome. Eur J Med Genet. 2016;59(9):444-51.

17. Tabebi M, Charfi N, Kallabi F, Alila-Fersi O, Ben Mahmoud A, Tlili A, et al. Whole mitochondrial genome screening of a family with maternally inherited diabetes and deafness (MIDD) associated with retinopathy: A putative haplotype associated to MIDD and a novel MT-CO2 m.8241T>G mutation. J Diabetes Complications. 2017;31(1):253-9.

18. Mezghani N, Mnif M, Kacem M, Mkaouar-Rebai E, Hadj Salem I, Kallel N, et al. A whole mitochondrial genome screening in a MELAS patient: a novel mitochondrial tRNA(Val) mutation. Biochem Biophys Res Commun. 2011;407(4):747-52.

19. Vargas-Poussou R, Houillier P, Le Pottier N, Strompf L, Loirat C, Baudouin V, et al. Genetic investigation of autosomal recessive distal renal tubular acidosis: evidence for early sensorineural hearing loss associated with mutations in the ATP6V0A4 gene. J Am Soc Nephrol. 2006;17(5):1437-43.

20. Romdhane L, Kefi R, Azaiez H, Ben Halim N, Dellagi K, Abdelhak S. Founder mutations in Tunisia: implications for diagnosis in North Africa and Middle East. Orphanet J Rare Dis. 2012;7:52.

21. Paul A, Drecourt A, Petit F, Deguine DD, Vasnier C, Oufadem M, et al. FDXR Mutations Cause Sensorial Neuropathies and Expand the Spectrum of Mitochondrial Fe-S-Synthesis Diseases. Am J Hum Genet. 2017;101(4):630-7.

22. Maalej M, Tej A, Bouguila J, Tilouche S, Majdoub S, Khabou B, et al. Clinical, Molecular, and Computational Analysis in two cases with mitochondrial encephalomyopathy associated with SUCLG1 mutation in a consanguineous family. Biochem Biophys Res Commun. 2018;495(2):1730-7.

23. Lefèvre C, Jobard F, Caux F, Bouadjar B, Karaduman A, Heilig R, et al. Mutations in CGI-58, the gene encoding a new protein of the esterase/lipase/thioesterase subfamily, in Chanarin-Dorfman syndrome. Am J Hum Genet. 2001;69(5):1002-12.

24. Louhichi N, Bahloul E, Marrakchi S, Othman HB, Triki C, Aloulou K, et al. Thyroid involvement in Chanarin-Dorfman syndrome in adults in the largest series of patients carrying the same founder mutation in ABHD5 gene. Orphanet J Rare Dis. 2019;14(1):112.

25. Melki I, Lambot K, Jonard L, Couloigner V, Quartier P, Neven B, et al. Mutation in the SLC29A3 gene: a new cause of a monogenic, autoinflammatory condition. Pediatrics. 2013;131(4):e1308-e13.

26. Jaouadi H, Zaouak A, Sellami K, Messaoud O, Chargui M, Hammami H, et al. H syndrome: clinical, histological and genetic investigation in Tunisian patients. The Journal of Dermatology. 2018;45(8):978-85.

27. Rejeb MB, Bahloul E, Sellami K, Mesrati H, Jaouadi H, Boudaya S, et al., editors. Syndrome H: une série de 5 cas du sud tunisien. Annales de Dermatologie et de Vénéréologie; 2018: Elsevier.

28. CHOUK H, REJEB MB, BOUSSOFARA L, EL MABROUK H, GHARIANI N, SRIHA B, et al. Phenotypic Intrafamilial Variability Including H Syndrome and Rosai Dorfman Disease Associated With the Same c. 1088G> A mutation in the SLC29A3 gene. 2021.

29. Yahia WB, Aounallah A, Daadaa S, Saidi W, Bousoffara L, Mokni S, et al., editors. Premier cas tunisien de syndrome H avec mutation du gène SLC29A3. Annales d'Endocrinologie; 2015: Elsevier.

30. Isrie M, Breuss M, Tian G, Hansen AH, Cristofoli F, Morandell J, et al. Mutations in Either TUBB or MAPRE2 Cause Circumferential Skin Creases Kunze Type. Am J Hum Genet. 2015;97(6):790-800.

31. Chamkha I, Alila-Fersi O, Mkaouar-Rebai E, Aloulou H, Kifagi C, Hachicha M, et al. A novel m.12908T>a mutation in the mitochondrial ND5 gene in patient with infantile-onset Pompe disease. Biochem Biophys Res Commun. 2012;429(1-2):31-8.

32. Chkioua L, Khedhiri S, Turkia HB, Tcheng R, Froissart R, Chahed H, et al. Mucopolysaccharidosis type I: molecular characteristics of two novel alpha-L-iduronidase mutations in Tunisian patients. Diagn Pathol. 2011;6:47.

33. Laradi S, Monastiri K, Ferchichi S, Nabli N, Aouini Rea P, Ben Limam H, et al. [Clinico-biologic and molecular study of mucopolysaccharidosis in central and southern Tunisia]. Ann Biol Clin (Paris). 2001;59(1):100-4.

34. Ouesleti S, Brunel V, Ben Turkia H, Dranguet H, Miled A, Miladi N, et al. Molecular characterization of MPS IIIA, MPS IIIB and MPS IIIC in Tunisian patients. Clin Chim Acta. 2011;412(23-24):2326-31.

35. Aadam Z, Kechout N, Barakat A, Chan KW, Ben-Ali M, Ben-Mustapha I, et al. X-Linked Agammagobulinemia in a Large Series of North African Patients: Frequency, Clinical Features and Novel BTK Mutations. J Clin Immunol. 2016;36(3):187-94.

36. Gritli S, Omar S, Tartaglini E, Guannouni S, Fleming JC, Steinkamp MP, et al. A novel mutation in the SLC19A2 gene in a Tunisian family with thiamine-responsive megaloblastic anaemia, diabetes and deafness syndrome. Br J Haematol. 2001;113(2):508-13.

37. Boukhris A, Stevanin G, Feki I, Denis E, Elleuch N, Miladi MI, et al. Hereditary spastic paraplegia with mental impairment and thin corpus callosum in Tunisia: SPG11, SPG15, and further genetic heterogeneity. Arch Neurol. 2008;65(3):393-402.

38. Boukhris A, Stevanin G, Feki I, Denora P, Elleuch N, Miladi MI, et al. Tunisian hereditary spastic paraplegias: clinical variability supported by genetic heterogeneity. Clin Genet. 2009;75(6):527-36.
